# Individual and household attributes influence the dynamics of the personal skin microbiota and its association network

**DOI:** 10.1186/s40168-018-0412-9

**Published:** 2018-02-02

**Authors:** Marcus H. Y. Leung, Xinzhao Tong, David Wilkins, Hedwig H. L. Cheung, Patrick K. H. Lee

**Affiliations:** School of Energy and Environment, City University of Hong Kong, B5423-AC1, Tat Chee Avenue, Kowloon, Hong Kong

## Abstract

**Background:**

Numerous studies have thus far characterized the temporal dynamics of the skin microbiota of healthy individuals. However, there is no information regarding the dynamics of different microbial association network properties. Also, there is little understanding of how living conditions, specifically cohabitation and household occupancy, may be associated with the nature and extent (or degree) of cutaneous microbiota change within individuals over time. In this study, the dynamics of the skin microbiota, and its association networks, on the skin of urban residents over four seasons were characterized.

**Results:**

Similar to western cohorts, the individuals of this cohort show different extents of variations in relative abundance of common skin colonizers, concomitant with individual- and household-associated changes in differential abundances of bacterial taxa. Interestingly, the individualized nature of the skin microbiota extends to various aspects of microbial association networks, including co-occurring and excluding taxa, as well as overall network structural properties. Household occupancy is correlated with the extent of variations in relative abundance of *Propionibacterium*, *Acinetobacter*, and *Bacillus* over multiple skin sites. In addition, household occupancy is also associated with the extent of temporal changes in microbial diversity and composition within a resident’s skin.

**Conclusions:**

This is the first study investigating the potential roles household occupancy has on the extent of change in one’s cutaneous microbiota and its association network structures. In particular, we show that relationships between the skin microbiota of a resident, his/her cohabitants, and those of non-cohabitants over time are highly personal and are possibly governed by living conditions and nature of interactions between cohabitants within households over 1 year. This study calls for increased awareness to personal and lifestyle factors that may govern relationships between the skin microbiota of one individual and those of cohabitants, and changes in the microbial association network structures within a person over time. The current study will act as a baseline for future assessments in comparing against temporal dynamics of microbiota from individuals with different skin conditions and for identifying residential factors that are beneficial in promoting the dynamics of the skin microbiota associated with health.

**Electronic supplementary material:**

The online version of this article (10.1186/s40168-018-0412-9) contains supplementary material, which is available to authorized users.

## Background

A community of microbial life forms, constituting of bacteria, fungi, viruses, and parasites make up the commensal skin microbiota responsible for modulating the host immunity responses and preventing colonization and invasion by pathogens [1]. Various medical and allergic conditions are associated with alterations in one’s overall cutaneous microbial community and dysbiosis between specific skin colonizers [2–4], highlighting the importance of the microbiota in cutaneous health. Thanks to advances in sequencing technology, the scientific community has gained a wealth of information regarding the skin microbiota, including the biogeography of the skin microbiota, roles of host properties, activities, and the environment on cutaneous microbial communities, co-abundance and co-exclusion correlations between taxa, and changes of microbiota associated with health and disease [4–15].

Recent investigations have also reported on the temporal dynamics of the cutaneous microbiota [2, 10, 16–21], highlighting the highly personal nature of the skin microbiota across healthy individuals over weeks and months. Changes in the microbiota may also be associated with cutaneous or immunological conditions, or as responses to clinical interventions [7, 19]. Altogether, these studies reinforce the importance of understanding potential relationships between the environment, host characteristics, and microbiota variations over time, and whether microbiota changes over time is related to temporal variations in disease phenotypes and prognosis [7].

As with other ecosystems, microorganisms found on skin engage in co-abundance and exclusion relationships [22]. While correlation does not equate causation, microbial association networks can be analyzed to infer potential ecological relationships between community taxa, as well as relationships between taxa and the environment. Recent works have performed correlation network analyses to characterize potential relationships between specific taxa within the skin microbiota [5, 6, 22]. However, understanding association networks at the structural level provides additional information on how microbial associations as a whole respond to different human or temporal variables, potentially revealing how this global association framework changes, and the factors that may drive such changes over time.

Previous studies have illustrated the effects of cohabitation on the skin microbiota within individuals. Specifically, our previous work [5] recapitulates the discoveries of other studies [19, 23] that the cutaneous bacterial assemblage of cohabitants are more similar compared to non-cohabitants. While these works complement earlier reports describing the effects of lifestyles and living conditions on occupant skin microbiota [13, 15], no study has extensively and directly evaluated the potential roles of cohabitation and household occupancy in shaping the nature and extent of changes in the skin microbiota and its association networks within individuals over time. Given that cohabiting individuals and their microbiota may facilitate the transmission of microorganisms [24], a greater insight into the potential roles of household properties such as occupancy on an individual’s skin microbiota and its dynamics may be of clinical significance.

Therefore, following our previous skin microbiota studies involving a single time point [5, 6], we now provide an account of the skin microbiota (while the term microbiota commonly encompasses all microbial domains, the term is hereafter referred to as the total bacterial and archaeal community) and its dynamics, of 24 individuals within Hong Kong (HK) households over the period of 1 year. Different aspects of the nature and extent of microbiota dynamics, with a focus on the roles of cohabitation and household occupancy, are described. We show that while our observations are consistent with the individuality of the dynamics of cutaneous microbiota shown in previous works [10, 20], we extend this personalized property to the dynamics of microbial association networks. At the same time, we suggest that the extent of changes in different characteristics of the skin microbiota may be associated with the number of cohabitants an individual resides with. By focusing on the changes of multiple aspects of the cutaneous microbiota at individual and household levels, this study sets the stage for comparative analyses pertaining to microbiota dynamics between healthy individuals and those with various skin conditions, thereby potentially identifying personal, household, and related factors associated with healthy microbiota dynamics.

## Results and discussion

In this study, we first characterize and discuss the nature and degree of variability of various facets of the skin microbiota within healthy individuals. This is followed by a focus of how effects of cohabitation and residential occupancy may play roles in shaping the observed differences in the degree of temporal variability in one’s skin microbiota over time.

### Taxonomic and sub-genus overview of the skin microbiota over time

Top genera (i.e., those with average relative abundance of ≥ 1% across the entire dataset) are detected across all individuals and skin sites and comprise under 60% of the skin microbial community, reflecting the overall taxonomic diversity of the cutaneous microbiota (Additional file 1: Figure S1 and Additional file 2: Table S1) [25]. The top genera include *Enhydrobacter*, which has been detected in previous reports and is considered to be enriched on skin surfaces of Chinese individuals [5, 26–28], suggesting that this genus is a prevalent and stable skin colonizer in subjects of this cohort. Information is currently lacking on the ecological and physiological bases for the apparent enrichment of *Enhydrobacter* in the Chinese. However, an association between relative abundance of *Enhydrobacter* on skin and facial sebum level and hydration has been reported in a recent Asian study [28]. Further examination of genetic and/or lifestyle factors that may affect skin physiology (such as living conditions, environmental exposure, food intake, and use of cosmetic and sanitary products [11, 25, 29–31]) may help elucidate the physiology of this colonizer on skins of Chinese and other host populations.

We sought to examine the extent of change in relative abundance of a given genus within an individual over the four seasons. Here, the extent of change in relative abundance for a particular genus is expressed as its coefficient of variation (CV). Regardless of skin site, each individual presents a distinctive signature of CV for each of the top genera (Additional file 2: Table S1 and Additional file 3: Figure S2). Previously, it was revealed that the relative abundance and the variations (as expressed by the standard deviation) of microbial taxa within the skin microbiota of a western cohort exhibit a second-order relationship, where low- and high-abundance microbial taxa vary less than those that are moderately abundant [10]. In contrast, our HK cohort shows strong linear correlative relationships across individuals (Additional file 4: Figure S3). As no genus in our dataset represents an average of over 45% of any individual’s microbiota, our correlative relationship between relative abundance and standard deviation over time is expected to be different from the previous study, where the second-order relationship observed appears to be strongly driven by taxa detected in over 50% of a subject [10].

**Fig. 1:**
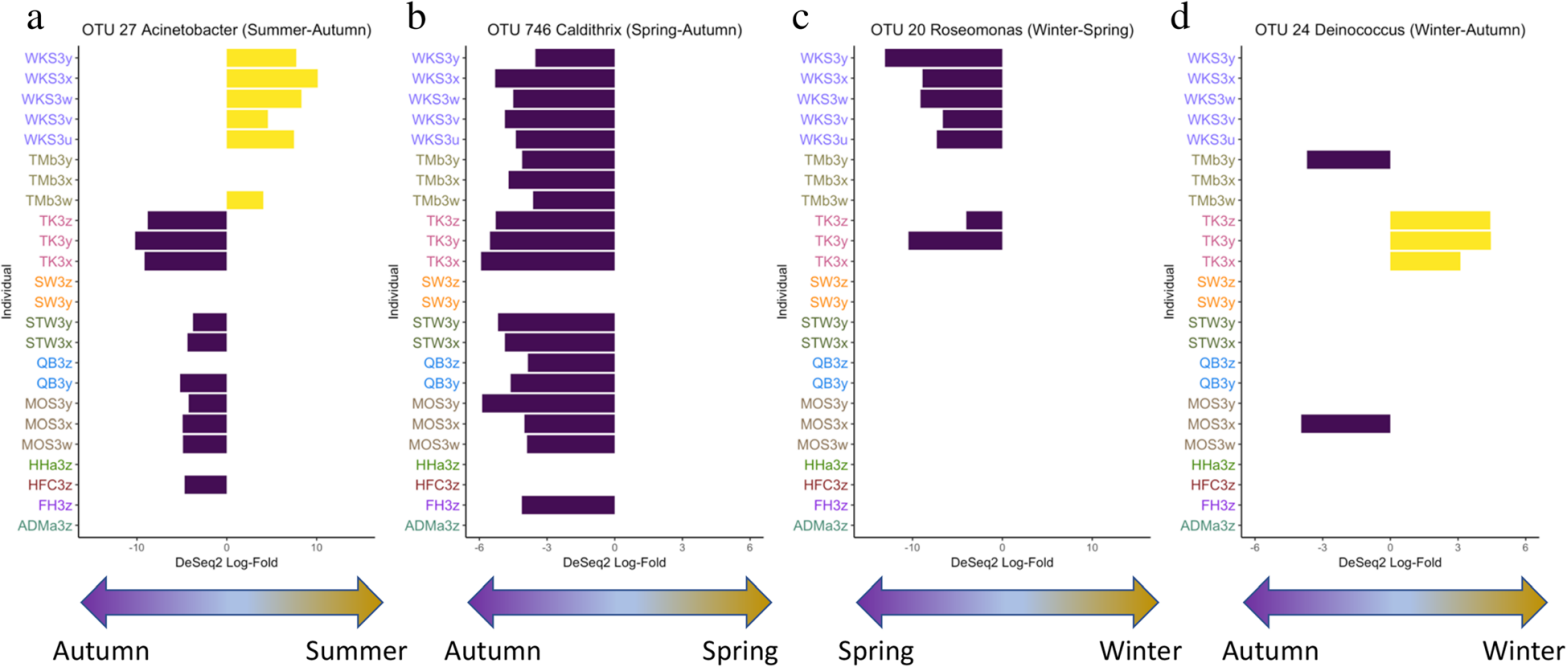
DeSeq2 differential abundance analysis of OTUs between pairwise seasonal comparison within individuals. OTUs showing cohort-wide differential abundances between seasons include **a** OTU 27 of *Acinetobacter* and **b** OTU 746 of *Caldithrix*. In addition, OTUs with household-specific differential abundance patterns include **c** OTU 20 of *Roseomonas* (household WKS) and **d** OTU_24 of *Deinococcus* (household TK). Note that despite **a** OTU 27 showing cohort-wide patterns, OTU 27 in occupants of WKS shows an opposite trend, showing a household-specific pattern in differential abundance. Occupants are color-coded according to households. A full list of OTUs with strong and significant differential abundances, which is defined by DeSeq2 log-fold difference of at least three and with an adjusted *p* value of ≤ 0.05, is presented in Additional file 5: Table S2

Differential abundance (Fig. 1 and Additional file 5: Table S2), negative binomial mixed model (Additional file 5: Table S2), and oligotype (Additional file 6: Figure S4) analyses reveal that genera are comprised of multiple species/strains, and specific taxa may be enriched in different seasons, independent of household or individual factors (for example, OTU_27 and OTU_746 in Fig. 1a, b; mixed model results are shown in Additional file 5: Table S2 and also oligotypes of genera in Additional file 6: Figure S4a-d). At the same time, taxa may be enriched in specific households/individuals within a season (for example, OTU_27 and OTU_20 within WKS, and OTU_24 within TK in Fig. 1a, c, d, and also oligotypes of genera in Additional file 6: Figure S4e-g). Cohort-wide variations in taxa distribution in different seasons may suggest general temporal trends in the emergence of particular species or strains on skin [10]. However, individual- or household-specific passive exposure may contribute to certain subjects or households carrying a particular species or strain at a given time point. It is possible that particular taxa may be picked up by cohabiting members via physical contact with residential surfaces, given the overlap between occupant skin and surface microbiota within households [21, 32, 33]. However, it is not known how these individual- or household-specific taxa can stably colonize the skin, as the fluctuations in relative abundance of taxa at different time points within an individual may also represent its poor adaptability and competitiveness with co-colonizers [10]. Whether a taxon presents population-wide or individual/household-associated temporal variation in abundance may be dependent on the taxon and how it interacts with the host, the environment, and co-colonizing microbes. As different species and strains may show variations in physiologies [34, 35], future temporal analyses of taxon-level variation in subjects may prove valuable in linking individual, household, and temporal factors with detection of taxa associated with increased virulence and resistance. Compared to the marker-based analyses, multi-omics strategies may provide more detailed genomic and metabolic assessments of strain variations between individuals, households, skin sites, and over time [9, 10].

### Microbial diversity variations within individuals over time

Shannon diversity, taking into account both community richness and evenness, was calculated to determine the nature and range of temporal variability in microbial diversity across individuals. When all samples were included for analysis, significant differences in Shannon diversity are observed between individuals, seasons, and skin sites (FDR-adjusted *p* ≤ 0.02 for all, Additional file 7: Table S3). Within each season, individuals and households are the major factors explaining significant differences in diversity. Linear mixed model also suggests that each of these major factors is potential drivers for differences in Shannon diversity (*p* < 0.001 for the three factors of season, individuals, and households. For each test, the other two factors, as well as sequencing batches, were considered as random variables). Significant differences in forehead and forearm diversities are observed between households, while forearms also exhibit significant differences in diversity between individuals (Additional file 7: Table S3).

To understand the personal rate of change in microbial diversity over the course of 1 year, we calculated the CV of Shannon diversity at a given site within an individual. Considering all samples, the Shannon diversity CVs are different between individuals, households, and occupancy (Kruskal-Wallis (KW) FDR-adjusted *p* < 0.005 for all). In contrast, CVs are not significantly different between samples of different skin sites, age group, and gender (FDR-adjusted *p* > 0.05, KW for skin site and age group, and Mann-Whitney (MW) for gender). Communities on left forearm sites varied in CVs between individuals and households (KW FDR-adjusted *p* < 0.04 for both, Additional file 8: Figure S5a-b). Shannon diversity CVs in forearms are also significantly higher in adults (average within-individual CV = 0.145) compared to the elderly (CV = 0.0871, KW post hoc pairwise *p* < 0.05, Additional file 8: Figure S5c).

### Microbial compositional variations within individuals over time

We analyzed variations in microbial community compositions between subjects, households, and within individuals over time using weighted UniFrac distances, taking into account both the presence and abundance of OTUs. Skin microbiota significantly cluster by season, individual, household, and age groups overall, as well as within separate skin sites, as shown by both analysis of similarities (ANOSIM) and permutational multivariate analysis of variance (PERMANOVA) analyses (Additional file 9: Table S4). Microbiota do not appear to cluster strongly by skin sites within each season in our cohort (and only marginally in summer).

Within an individual, the microbiota of forearm and palms are more similar within than between seasons (Additional file 9: Table S4 and Additional file 10: Figure S6. Forehead sites were not analyzed, as there is only one forehead sample per individual per season). Also, within an individual, the extent of microbiota dissimilarity on a skin site between any two time points is significantly and positively correlated with that of another skin site (Table 1), consistent with the work of Flores et al. [20]. Furthermore, extremity sites within individuals with greater extent of temporal change in microbial diversity also vary greater in the change in community composition over the year (Additional file 11: Figure S7). While correlations between temporal community variation and average community diversity (i.e., beta-diversity versus alpha-diversity) within an individual over time have been reported previously [10, 20], here, we demonstrate that the extent of changes in the two community properties (beta-diversity versus CV of alpha-diversity) are also correlated.

**Table 1:** Spearman’s correlation based on pairwise weighted UniFrac distance within individual over time

	Forehead	Left forearm	Right forearm	Left palm	Right palm
Forehead^a^		0.479	0.268	0.314	0.372
Left forearm			0.611	0.542	0.454
Right forearm				0.525	0.467
Left palm					0.539

^a^All correlations statistically significant (FDR-adjusted *p* < 0.001)

As with previous investigations [10, 17], the microbiota of the same site within some individuals between seasons closer together are more similar, as suggested by the significantly negative time-decay relationships (Additional file 9: Table S4). As samples for this study were collected within a single year, it is currently not known whether the decay relationships observed will persist for longer time frames as in other ecosystems [36], or whether an annual or seasonal cyclical pattern will emerge over longer periods of time, as seen in the gut microbiota of different mammals [37] and potentially in humans [38]. A greater understanding of time-decay relationships of cutaneous microbiota has forensic implications, where the identifiability of a person’s microbiota on a surface may decrease over time [21]. Given that time-decay relationships are more likely to be a personal trait rather than one shared by members of a cohort or household level (based on our results here and those reported previously [20]), additional works will be required to understand why some subjects present time-decay relationships and some do not, and how inter-individual time-decay variability affects the feasibility of using microbiota data in forensic applications.

Overall, our observations are congruent with previous western studies looking at the temporal dynamics of the skin microbiota [10, 17, 20]: (1) individuals experience ranges in the extents of temporal changes in relative abundance of genera and overall microbial diversity and community composition, (2) time-decay relationships in microbiota are present but not a ubiquitous property on skin, and (3) within the same individual, multiple skin sites present significant correlations in the extent of microbiota variations at any two time points, and (4) there exists significant correlations between the extent of temporal variations between different skin sites. Therefore, despite known differences in microbial community compositions across continental populations [5, 11, 13–15, 30], some temporal properties of cutaneous microbiota may be more universal. Such knowledge regarding the behaviors of microbial communities within and between individuals over time may provide fundamental basis for subsequent and more focused microbiota analyses in special populations around the globe, such as those adopting different lifestyles and are living in drastically different environments [13].

### Dynamics of microbial networks are individualized in associating taxa and overall network structure

While the dynamics of microbial diversity and community composition within individuals have been examined above and in previous works [7, 10, 17, 20], little information is currently available on the temporal properties of microbial correlative association networks. As correlative association may represent ecologically significant relationships between microbial constituents, tracking correlations over time in each individual will allow the assessment on the factors governing network structure property at a personal level. Here, insights into SParse InversE Covariance Estimation for Ecological Association Inference (SPIEC-EASI) [39] were used to (1) identify co-abundance and co-exclusion relationships between specific taxa within individuals and (2) determine whether within-individual network structure dynamics can potentially be explained by individual, household, or temporal factors.

**Fig. 2:**
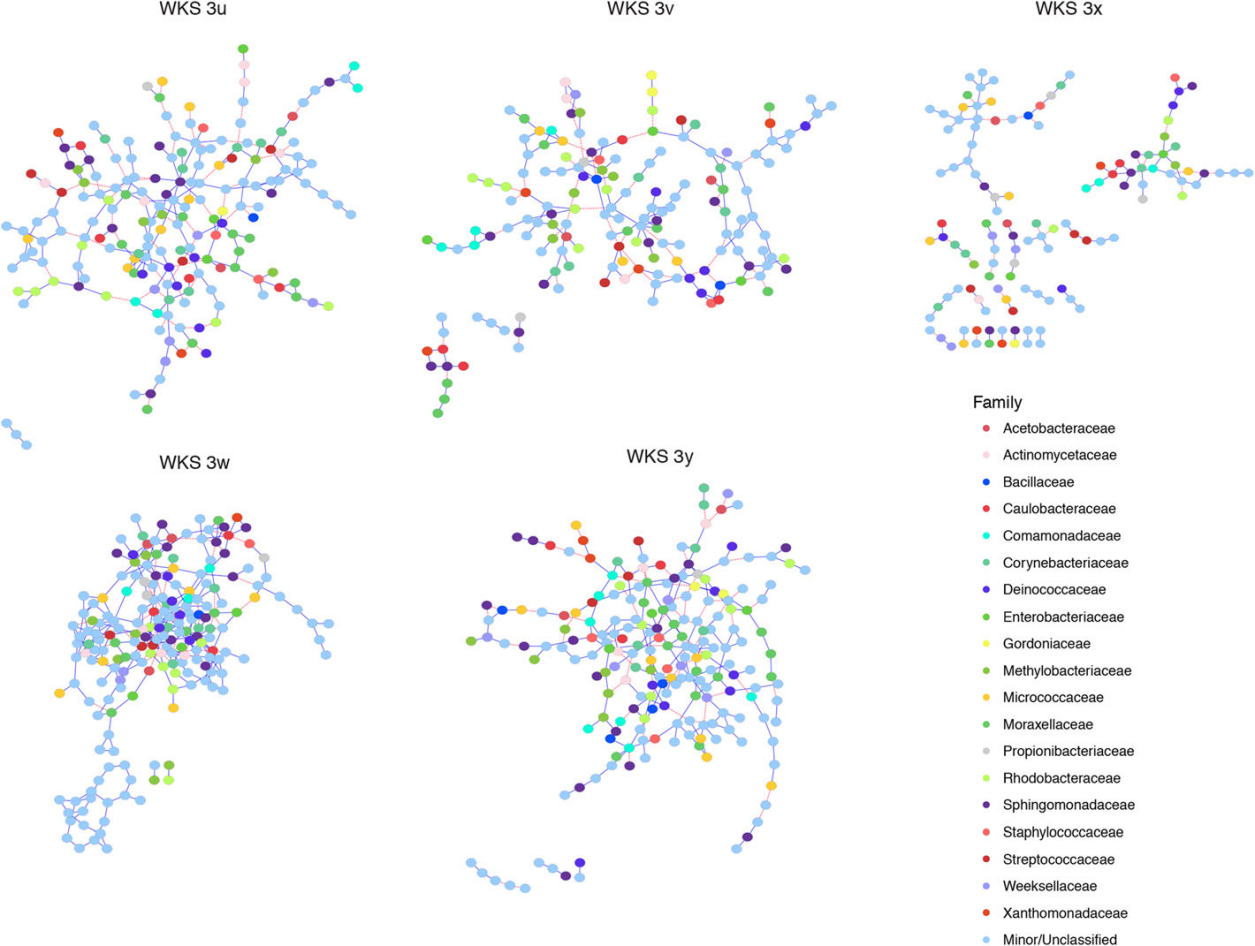
Microbial association network for individuals of household WKS over the period of 1 year. Significant correlative associations between OTUs (represented by nodes) within each individual determined based on the SPIEC-EASI pipeline. Correlations between OTUs can be positive (represented by blue edges) or negative (represented by red edges). SPIEC-EASI correlations with magnitude of < 0.05 are not represented in figure. OTUs belonging to one of top 20 taxonomic families are color-coded, whereas OTUs of other families are grouped into “ minor/unclassified” group, represented by dark gray nodes. Networks of individuals within WKS presented here as a visual example of the differences in network structures. Network representations of remaining individuals are included in Additional file 12: Figure S8, and a full list of significant correlative associations is presented in Additional file 13: Table S5

Within an association network for each individual over the course of 1 year (networks of individuals in WKS are presented as examples in Fig. 2; networks of remaining individuals in cohort are presented in Additional file 12: Figure S8, and Additional file 13: Table S5), positive correlations represent an average of 82.2% (71.0–92.2% for 24 individuals) of all associations, consistent with previous works demonstrating that the majority of significant correlations between skin sites are positive [22, 40]. Intra-genus correlations OTU pairs are more likely to be positive, whereas OTUs of different genera may be involved in both positive and negative correlative associations (Fig. 2, Additional file 12: Figure S8, Additional file 13: Table S5, and Additional file 14: Figure S9). A taxonomically diverse collection of OTUs are involved in significantly strong associations, suggested by the large number of OTU nodes classified as “minor/unclassified.” Moreover, most of the highly connected nodes (i.e., key hub OTUs) are not necessarily the most abundant taxa (Additional file 13: Table S5). Relatively minor and rare microbial members within the microbiota have been appreciated as possibly important drivers of community composition dynamics [41]. Here, we complement our previous works demonstrating that these rare taxa are not merely present on skin [5], but may engage in correlative relationships with other microbial members, and are potentially central members in the maintenance of association network structure [42]. Individuals within a residential unit do not share similar hub OTUs, suggesting that microbial members central to association structures are not strongly influenced by household factors.

While strong positive associations are detected within OTUs of *Enhydrobacter* across individuals of different households (QB3z and SW3z), this genus is also involved in positive associations with OTUs of other genera (OTU_4 of *Enhydrobacter* with OTUs of *Chryseobacterium* and *Acinetobacter* within households of STW and TMb, respectively Additional file 14: Figure S9). Consistent with our previous work [5], *Enhydrobacter* is negatively associated with OTUs of *Streptococcus* (HFC3z, Additional file 14: Figure S9). OTUs of *Enhydrobacter* and *Corynebacterium* can be involved in both positive and negative associations (within individual TMb3x), demonstrating that species and strains of *Enhydrobacter* and other genera deserve further analyses in not only its physiology on skin but also their potential interactions within the skin microbiota.

With the exception of individual MOS3x, most of the associations between OTUs of *Propionibacterium* and *Staphylococcus* are positive correlations. *Propionibacterium acnes* and *Staphylococcus epidermidis* (the dominant Staphylococcal species on skin) can be mutually inhibitory, but genetic variations between strains influence the extents to which one species affect the physiology and survival of the other [43]. Previous species-level analysis of *Staphylococcus* in our cohort reveals that as detected in some healthy individuals in the western world [18], most OTUs of this genus are in fact *Staphylococcus aureus* [5]. Intriguingly, *S. aureus* has been shown to respond to molecules secreted by *P. acnes* at particular conditions [44]. While correlation analyses act as gateways for identifying interactive relationships between microbial members, it provides no information on the environmental and strain physiological states necessary for such interactions to occur. Coupled with the recent identification of separate evolutionary patterns between continental strains of *S. aureus* [45], the results here again beckon the need for shotgun metagenomics sequencing to examine microbial associations at higher resolution, not only to understand species- and strain-level patterns in microbial associative relationships but also the metabolic and physiological bases for such relationships [10].

**Fig. 3:**
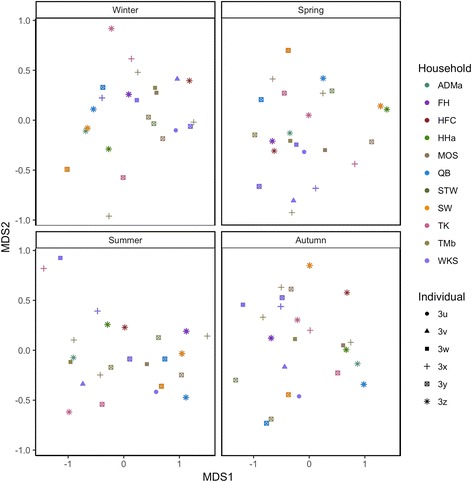
Multidimensional scaling plot (MDS) of graphlet correlation distances between microbial association networks of individuals over four seasons. Network structures (each network represented by a data point) in each season that are closer together along the MDS axes have more similar association graphlet structures. The MDS result is separated into four plots for visual ease, and MDS axes are identical in the four plots, as ordination was performed with data points from all four plots included for analysis. Points are color-coded by household and shaped according to individuals within the households

Following the description of taxa within association networks, we assessed the dynamics of association network structures within individuals over the four seasons by generating a network for each season within every individual. Graphlet correlation distance information between network pairs was used to visualize similarities in graphlet properties between association networks in a multidimensional scaling (MDS) plot (Fig. 3). Graphlet MDS ordination suggests that the dynamics of the association networks are highly personal and do not appear to be governed by seasonal or household factors considered in the study. Similarly, the dynamics of the association network structure, as measured by node degree distribution (Additional file 15: Figure S10a) and natural connectivity (Additional file 15: Figure S10b-c), also appear to be individualized. Microbial association network structures have been analyzed and compared between different natural and human ecosystems [5, 22, 39, 40, 46], but not between individuals, nor over multiple time points within individuals. Here, we surmise that the temporally personal nature of the human skin microbiota is not merely limited to its community diversity and composition [10, 20] but extends to the potential ecological interactions within its members within this microbiota. Whether these personal differences in association network dynamics play roles in differences in susceptibilities to conditions associated with skin microbiota dysbiosis is unclear, but our association network findings here necessitate a more individual and egocentric approach in the future by linking personal attributes to microbial associations, and whether the nature of changes in association networks over time can act as indicators or predictors for cutaneous health and disease. Our observations here also raise the question of, despite the diversity observed across individuals and over time, whether a core microbial association network for the skin microbiota exist across healthy individuals at a time and across time within an individual [22], and how this core network is affected by the overgrowth of opportunistic pathogens associated with disease onsets [18]. These valuable questions should be addressed in future large-scale skin microbiota investigations.

### Roles of cohabitation on microbiota changes within individuals over time

**Fig. 4:**
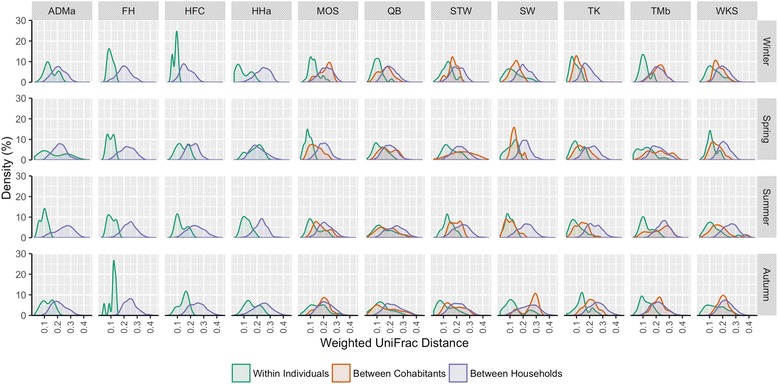
Density plots of pairwise UniFrac distances between samples of the same individuals (green), between cohabitants (orange), and between non-cohabitants (purple) across seasons. Density plots are faceted by household and season. Households with data from only one occupant (ADMa, FH, HFC, HHa) do not have cohabitant curves

Microbiota within individuals are more similar than those between non-cohabitants regardless of season (Fig. 4 and Additional file 16: Table S6), which is similar to our previous study [5] based on a single time point and conglomerating all subjects as a unit. However, depending on the individual and/or time point, the similarity in microbiota between a resident and his/her cohabitant may not be significantly greater than that between that resident and non-cohabiting individuals (by post hoc test indicating no significant differences in average pairwise weighted UniFrac distances between cohabitants and between households for a particular individual, Additional file 16: Table S6). Conversely, in other instances, the microbial assemblage within a resident is not significantly more similar than that between his/her cohabitants (by post hoc test indicating no significant differences in average pairwise weighted UniFrac distances between within individual and between cohabitants, Additional file 16: Table S6). Therefore, the effects of cohabitation on microbiota similarities between individuals may be more personal and complicated than previously appreciated. Song et al. [23] report that cohabiting couples share more similar microbiota compared to between parents and their children and that dogs may be a factor in homogenizing skin microbiota between cohabitants. In contrast, a more recent study shows lack of microbiota differences between cohabitants of different relationships [19]. Our cohort do not present enough individuals to assess how particular relationships between household members affect individual microbiota over time, and how domesticated animals contribute to the overlapping of microbiota between cohabitants over time. Nonetheless, given the lack of consistency over seasons in how more or less similar cohabitants’ microbiota are between different individuals and households in our cohort, we hypothesize that similarity between microbiota of cohabitants is more complex than the cohabitants’ relationships alone. Alternatively, specific interactions between cohabitants, differences in skin physiologies, and lifestyle [23, 25] between different occupants within a household likely play more prominent roles in determining each occupant’s microbiota, how variable the microbiota is within the occupant, how similar the microbiota of his/her cohabitants are, and whether such similarities change over time.

**Fig. 5:**
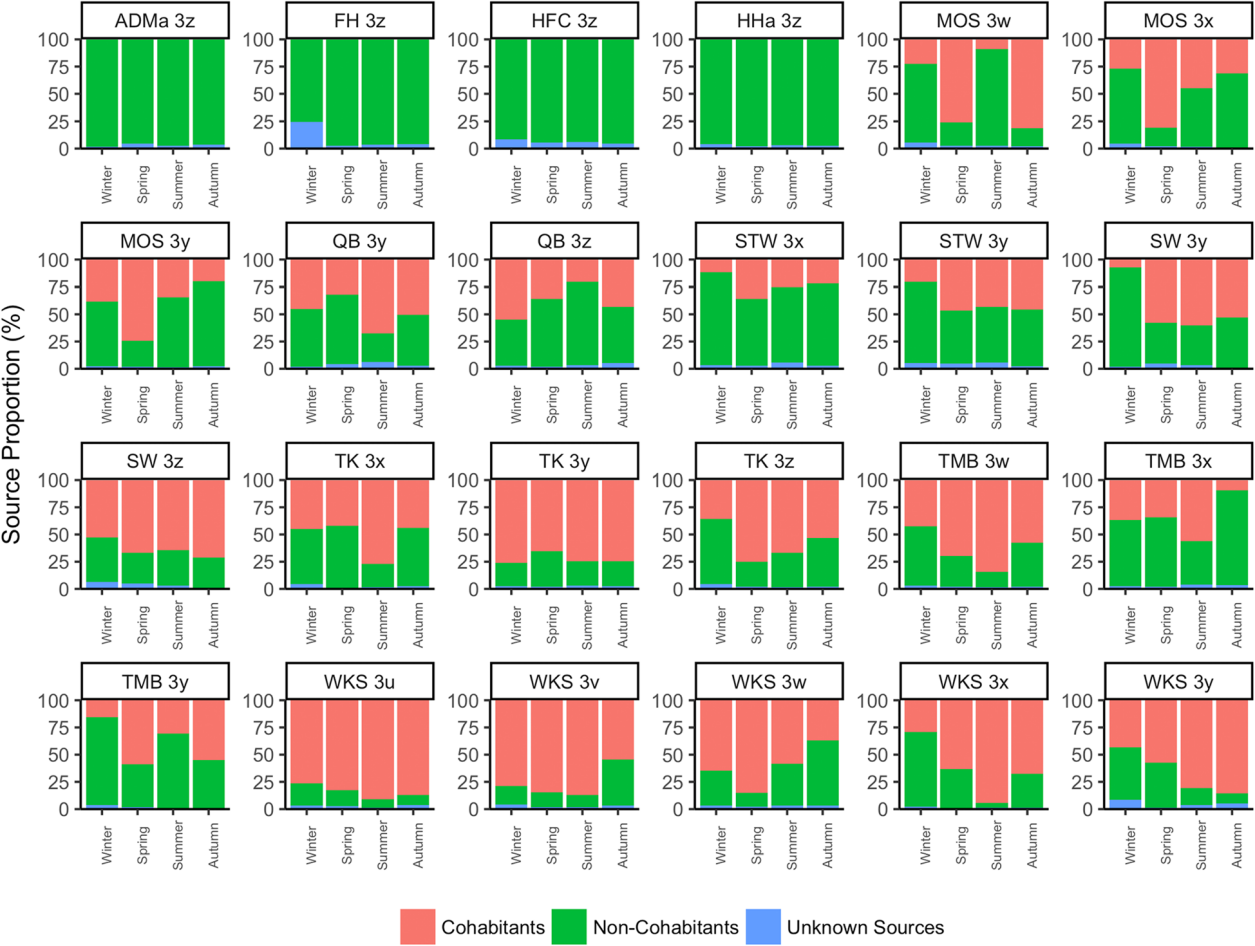
Estimated proportions of cohabitants’ skin microbiota as potential sources of an individual’s skin microbiota. Source prediction are grouped by individual and season. For a given individual, a microbiota source can be from cohabitants (red) or from non-cohabitants (green). Individuals from households ADMa, FH, HFC, and HHa either live alone or do not have cohabitant microbiota data. Microbiota that cannot be sourced with confidence are indicated as “Unknown Sources” (blue). Source proportion estimated using the Bayesian SourceTracker analytic tool [47]

The contribution of cohabitants’ skin microbiomes as potential sources of an individual’s microbial assemblage in each season was assessed using Bayesian SourceTracker analysis [47]. For individuals with cohabitants (i.e., excluding households ADMa, FH, HFC, and HHa where no cohabitant microbiota data is available), the proportions of each subject’s microbiome coming from cohabitants range from 7.0% (SW3y sink in winter) to 94.2% (WKS3x sink in summer) (Fig. 5). No one season appears to be associated with an increased sourcing of individuals’ microbiomes from cohabitants across households, suggesting that the potential effects of cohabitants in sourcing one’s skin microbiome has minimal temporal effect within the year of sampling. In contrast, the overall proportions and dynamics of cohabitants’ microbiome as potential sources of each individual’s skin microbial community are personal in nature. In addition, there is an overall significant and positive correlation between the proportion of one’s skin microbiome potentially sourced from cohabitants and the number of cohabitants that sink subject is living with (Kendall’s *τ* = 0.210, *p* = 0.01). However, this can merely be due to the increased number of cohabitant samples as sources available for analysis. Nonetheless, in all households, the proportion of unknown sources is low (average proportion of 3.3% within an individual at a given time point). This suggests that the microbial assemblages across our subjects overall are relatively similar, and any individual- and household-based differences may be due to microbial taxa that are low in abundances. Given that this cohort is a relatively small collection of cohorts within a single city, a similar analysis can be performed in cohorts across continents to test whether global skin microbiota, which are known to present community differences [5, 11, 13–15, 30], present similar extents of source overlap over time, so as to potentially infer the size of the global skin pan-microbiota [5, 48].

### Roles of occupancy on degree of microbiota changes within individuals over time

Members within a household do not appear to share similar signatures of CV in relative abundance of specific genera, depending on the genus and skin site. However, significant correlations can be observed between CVs of relative abundance and household occupancy. Specifically, individuals within households of higher occupancy are associated with greater CVs of *Propionibacterium* and *Acinetobacter* over the period of the year (Additional file 17: Figure S11a-c). Conversely, a significantly negative correlation, as shown for *Bacillus* on palms, suggests that individuals with fewer cohabitants tend to have greater rates of changes in relative abundance of this genus (Additional file 17: Figure S11d). In addition to changes in relative abundance of specific genera, individuals residing in households with higher occupancy also present higher CVs of Shannon diversity on left forearm but not for the right forearm and other sites (Additional file 17: Figure S11e). The significance on the left forearm is intriguing and may perhaps be related to the combined effects of handedness (since all but one individual in this study are right-handed) and environmental exposure. However, this is speculative, and additional investigations in controlled settings will be required to assess the roles of these effects, and with a focus on how cohabitation, handedness, and environmental exposure affect the temporal dynamics of skin microbiota. Thus, it is currently not known whether the different correlation trends in different skin sites are ecologically significant. Metagenomic shotgun sequencing may prove beneficial in explaining any potential ecological and physiological rationale for the correlative observations here.

To examine whether the number of cohabitants within a household is also associated with variations in community composition of the skin microbiota of each occupant and site, Kendall *τ* correlation was performed between the mean UniFrac distances for a particular site within an individual between any two seasons, and the number of occupants present in his/her household. Forehead and left forearm sites from individuals living in residences of higher occupancy show more temporally variable microbial communities over the course of the year (Additional file 17: Figure S11f).

Overall, the number of residents within a household appears to be associated with changes in the dynamics of the personal skin microbiota over 1 year at multiple levels: (1) the extent of variation in the relative abundance of specific colonizers (CVs of *Propionibacterium*, *Acinetobacter*, and *Bacillus* relative abundance across multiple skin sites), (2) the variation in community diversity (as determined by CVs of Shannon diversity within each individual on forearm sites), and (3) the variation in community structure (through average weighted UniFrac distances between forehead and left forearm sites of the same individuals over seasons). Our households are limited to a maximum number of six occupants, and it is currently not known how residences with more individuals, or other built environment settings (such as workplaces and other public spaces of higher occupancy) would affect one’s skin microbiota and its dynamics.

However, it is unlikely that occupancy directly shapes microbiota changes within occupants, as individuals living in different households of the same occupancy still have different microbiota and variable CVs. Instead, we propose that the number of residents within a household indirectly affects an occupant’s microbiota dynamics by how an occupant interacts with the cohabitants and their microbiota. A household with more people may facilitate increased contact, which may facilitate direct transfer of personal skin taxa and potentially pathogenic microorganisms [12, 24]. Alternatively, occupants may release personal microbiota into the residential air and onto surfaces, and microbial members may subsequently be picked up by its cohabitants through passive exposure or active surface touching [21, 32]. We suggest that over single or multiple time points, the relationships between one’s own microbiota and that of its cohabitants and others (that is, how similar or different their microbiota are, Fig. 4 and Additional file 16: Table S6) depend on the biogeographical (across skin sites within a time point) and temporal (within a site over multiple time points) variability of a person’s microbiota (which is in itself now known to be highly personal [10, 20]) and the nature of interactions between the person and his/her cohabitants. Individual factors, in addition to household occupancy and cohabitation, may as a result influence the temporal stability of one’s baseline microbiota over time, which is consistent with our pairwise UniFrac (Additional file 16: Table S6) and SourceTracker (Fig. 5) observations. Therefore, occupancy and cohabitation represent part of a collection of household properties, along with living conditions and lifestyles [15] that may influence the nature and the extent of change in one’s skin microbiota over time. In addition, given that population density in HK is among the highest in the world, it is currently not known how household properties that take into account the crowdedness of households (such as occupant density or privacy indexes as defined previously for microbiota of residences [49]) will influence skin microbiota of residents. We believe that separating these household properties, perhaps via controlled chamber and inoculation experiments [7, 17], will be crucial in specifying the relative proportion of contribution of each household factor in shaping an individual’s microbiota dynamics.

## Conclusion

While our findings corroborate previous western studies in identifying the personalized properties of skin microbiota dynamics, we have highlighted in this study possible associations between occupancy to multiple aspects of microbiota dynamics within individuals, including for the first time, the changes of microbial association network structures within every individual over 1 year. Our findings underscore the need for close examination on individual physiological and lifestyle properties in explaining changes in cutaneous microbiota but also an in-depth assessment of the person’s living environment, including number of cohabitants and their interactions over time. Also, while a daunting task, a more individualistic approach may therefore be beneficial to determine factors governing changes in microbial association network structures within an individual, thereby gaining understanding on properties associated with network dynamics associated with adverse skin conditions.

Finally, there is little information regarding whether individuals suffering from cutaneous ailments present different temporal patterns on their skin microbiota from that of healthy individuals, in ways similar to how gut microbiomes of individuals suffering from various gut conditions have distinctive microbiota temporal traits [50]. Data presented here therefore can act as community-level baseline in understanding how the microbiota dynamics of healthy individuals compare with those with various skin conditions. Future longitudinal and comparative microbiota works will potentially provide perspectives on whether disease onset, progression, and recovery are related to changes in microbiota-host relationships over time and identify host and other factors that may be associated with these changes.

## Methods

### Cohort characteristics and sampling

A total of 480 skin samples from this study are part of a larger seasonal analysis of skin, air, and surface microbiomes of Hong Kong residences [21], and a continuation of previous single-season skin microbiota works [5, 6]. Ethics approval for subject sampling was granted by the City University of Hong Kong Ethics Committee (reference number 3-2-201,312 (H000334)). After being informed about the nature of the study, as well as their roles and responsibilities as subjects, written informed consent was given by all individuals.

From 24 healthy individuals (coded one of 3u to 3z) of 11 households (ADMa, FH, HFC, HHa, MOS, QB, STW, SW, TK, TMb, WKS), samples were collected in January (winter), April/May (spring), August (summer), and November/December (autumn) of 2014 (Additional file 18: Table S7). All samples were collected between 19:00 and 20:00, as individuals were most likely to be at home during those times and to minimize the disruption caused to the individuals due to sampling. For each individual, five skin samples (forehead, left/right forearm, left/right palm) were collected at his/her residence by samplers wearing gloves sterilized with 70% ethanol to minimize contamination or skin taxa transfer between sampler and the subject. All subjects included this study are ethnically Chinese and are long-term residents of Hong Kong. Subjects of this study had not taken antibiotics and antifungals at least 3 months prior to each sampling episode. The individuals in this study were living in households throughout rural and urban areas of Hong Kong to cover a broad local geographical scope. Individuals and households were selected to cover a range of age and lifestyle choices such as smoking, pet ownership, and allergies. All households involved in this study did not use pesticide or have purchased new furniture during the course of the sampling periods. While antibiotic ingestion may or may not have an effect on skin microbiota dynamics [19, 20], we note that all subjects had not taken antibiotics 1 month prior and after each sampling period. Subjects were instructed to not wash their hands or shower immediately prior to sampling. Samples were collected as previously described [5]. Briefly, skin samples were collected using sterile cotton swabs moistened with 100 μL swab solution (0.15 M NaCl and 0.1% Tween 20) [25], and each sample was collected using a back-and-forth swabbing motion for 15 s. Samples were stored in − 80 C within 1 h of sampling and were stored until genomic DNA (gDNA) extraction.

### Genomic DNA extraction, 16S rRNA gene amplification and sequencing

Following sampling, gDNA was extracted using the PowerSoil DNA Isolation Kit (MO BIO Laboratories, Inc., Carlsbad, CA, USA), with modifications as described previously [5]. Sterile cotton swabs not exposed to skin surfaces were included in the extraction process as negative controls to account for possible contamination. Purified gDNA samples, including negative controls, were sent to Health Genetech Corporation (Taoyuan City, Taiwan) for PCR, library preparation, and sequencing. PCR, library preparation, and Illumina MiSeq sequencing were prepared as described [5, 51]. Briefly, the 515f/806r primer pair was used to target and amplify the V4 hypervariable region of the 16S rRNA gene [29]. PCR amplification was performed in a 20-μL reaction volume containing 10 μl 2× Phusion HF master mix (New England BioLabs, Ipswich, MA, USA), 0.5 μM each forward and reverse primer, consisting of customized barcodes present on both primers for multiplex sequencing, and 50 to 150 ng DNA template. The PCR conditions consisted of an initial of 98 °C for 30 s, followed by 30 cycles of 98 °C for 10 s, 54 °C for 30 s, and 72 °C for 30 s, as well as a final extension of 72 °C for 5 min. Positive amplification was verified by agarose gel electrophoresis. Amplicons in triplicates were pooled and purified using AMPure XP beads (Agencourt, Brea, CA, USA) and quantified using a Qubit double-stranded DNA HS assay kit on a Qubit fluorometer (Invitrogen, Carlsbad, CA, USA), all according to the respective manufacturers’ instructions. For library preparation, Illumina adapters were attached to amplicons using the Illumina TruSeq DNA sample preparation kit v2. Purified libraries were applied for cluster generation and sequencing on the MiSeq platform using paired-end 150-bp reads.

### Sequence analysis

A total of 12,212,096 forward sequences in .fastq format underwent quality filtering using the “fastq_filter” command in USEARCH [52], based on a maximum expected error of 1 error/read, reads trimmed to a length of 139 bp and shorter reads removed. Eighty percent of reads (9,792,397 reads) were of acceptable quality. Filtered reads were then de-multiplexed and binned into OTUs using UPARSE with 97% sequence identity threshold [53]. OTUs were provided with taxonomic information using the “assign_taxonomy.py” command in QIIME [54] based on the Greengenes reference database (May 2013 version, 99,322 sequences). Chimeras were detected in UCHIME2 (high-confidence mode) against the Greengenes database to minimize risk of identifying false-positive chimera OTUs [55]. OTU lineages present in an average of > 5% of reads in negative controls (each control averages 20,000 reads and 600 OTUs) were considered contaminants and were removed from all samples. OTUs classified as chloroplasts and mitochondria were also removed. Following read filtering and OTU removal, a total of 8,093,026 reads are retained for community analyses described below. Following quality control and OTU taxonomic classification, archaeal OTUs represent ~ 0.3% of reads detected.

### Bioinformatics analyses

The coefficient of variation (CV) of relative abundance for top genera for each individual over the four seasons is calculated by the standard deviation of the genera within that individual, divided by the mean of the relative abundance of that genera in the individual. A low CV represents a relatively stable relative abundance for that given genus on a given individual, and high CV represents less stable relative abundance. Total Shannon (alpha-) diversity for each sample was estimated using the “breakaway” package (version 4) in R available from CRAN [56]. Shannon diversity values presented in Additional file 7: Table S3 are average values following 999 reiterations. Similar to previous skin microbiota works [10, 20], the CV for Shannon diversity within each individual, representing the change of within-sample microbial diversity of an individual over the four seasons, is calculated by the standard deviation of the Shannon diversity values for that individual, divided by the mean of the Shannon diversity of the same individual. Weighted UniFrac distances were computed using the QIIME script “beta_diversity.py.” Analyses of similarities (ANOSIM) were computed on based on β-diversity data using “vegan” package in R. The PERMANOVA pseudo-F statistic and significance were calculated using QIIME’s “compare_categories.py” script (with 999 permutations) based on the weighted UniFrac distance matrix generated from QIIME. Differential abundance of OTUs between sample groups were performed with DeSeq2 using the QIIME script “differential_abundance.py,” performed separately for each individual and each season pair. OTUs that showed significant DeSeq2 results in more than half of the individuals for a given season pair were included for negative binomial and zero-inflated negative binomial mixed model analyses (“glmmADMB” package in R) to confirm significant differences in abundance for these OTUs, using season as a fixed effect and count data as response variable. Variations between individuals, skin sites, and sequencing batches were considered as random effects. OTUs that appear in less than 20% of samples, and OTUs with less than 100 reads were not included for the analysis. OTUs of top genera that were detected at an average of ≥ 1% relative abundance and were present across all samples were subjected to oligotyping (version 2.1 from http://merenlab.org/software/oligotyping/) [57]. To remove oligotypes with low read counts, a minimum substantive abundance (*M*) of 10 was adopted. A linear mixed effects model using the R package “lme4” was used to determine whether Shannon diversity differences can possibly be driven by seasonal, individual, and household factors, with individual, site, household, and/or sequencing batch differences as random effects when not considered as a predictor effect. The analysis of variance (ANOVA) on the hypothesized and null models was performed to determine statistical significance in Shannon diversity differences for each predictor factor.

### Microbial source-tracking analysis

To examine the roles of cohabitants as potential microbial sources of an occupant’s skin in each residence and time point, Bayesian SourceTracker [47] analysis was used to estimate the proportional contribution of the cohabitants’ skin microbiome to the individual microbiome at the given season. OTUs present in less than 10% of the samples were excluded for the analysis. SourceTracker predictions were generated for each individual across four seasons, with the skin samples of each individual in one season as the sinks, and the skin samples of the remaining individuals in the same season as potential sources. The results were presented as the source proportions from cohabitants, non-cohabitants, and unknown sources based on whether the sink and source communities can be sourced from the same household.

### Microbial association network analysis

SParse InversE Covariance Estimation for Ecological Association Inference (SPIEC-EASI) was used to assess potential ecological associations between microbial taxa. SPIEC-EASI has been recently applied to other microbiota investigations [58, 59] and is currently one of the preferred methods for association network analysis, as it is robust to issues of compositional bias, conditional independence, and dimensionality, all properties commonly encountered in microbiota data [39]. Two sets of association network analyses were performed, where (1) microbiota data for all seasons within an individual was combined (to assess for overall microbial associations within an individual, set of 24 networks), and (2) where microbiota data for each season within each individual was analyzed separately (to assess for dynamics of association networks within individuals, set of 96 networks). For each group, abundance data over different samples underwent centered-log ratio transformation. OTUs with less than 150 and 10 reads were not included in the first and second analysis, respectively. Based on transformed data, the Stability Approach to Regularization Selection (StARS) method [60], suitable for high-dimensional data, was used to infer the network structure, employing the node-based neighborhood selection procedure, with a minimum lambda ratio of 0.01 and reiteration of 50 times as recommended [39]. Networks as shown in Fig. 2 were constructed with Cytoscape (version 3.4.0) [61].

Using SPIEC-EASI, general network structural attributes, including degree distribution (distribution of the number of significant associations (i.e., edges) per each OTU (i.e., node), and natural connectivity (a proxy for network stability and robustness to targeted node removal, by assessing the extent of alternative paths present between any two nodes) for each network were also determined. Natural connectivity for network within each individual and season was assessed based on node removal of either decreasing node betweenness centrality, which is a measure of the paths between a node to all other nodes, or decrease node degree, which is a measure of the number of edges a particular node contains. Natural connectivity is presented as the proportion of the total network size, and a higher natural connectivity represents a more stable and robust association network upon node removal. Graphlet correlation distance approach as described by Yaveroğlu and colleagues [62] was also performed on the set of 96 networks to compare the roles of season, household, and individuals in shaping association network structure. Briefly, each network was broken down into subgraphs (graphlets), each consisting of up to four nodes, and performed the graphlet frequency distribution of each node across networks. A graphlet correlation matrix was generated for each of the 96 networks based on pairwise Spearman correlations of the 11 non-redundant orbits of all nodes. This matrix is then used to calculate the graphlet correlation distances, and relationships between networks which were then visualized into multidimensional scaling plots constructed using R. Each network is represented by a position on the plot, and networks that are closer to each other in the MDS plot space are considered to be more similar in network structure.

### Statistical analyses

The nonparametric Mann-Whitney (MW) and Kruskal-Wallis (KW) tests were employed to determine significance when comparing between two or more comparison groups, respectively. *p* values were adjusted for multiple comparisons using the false-discovery rate (FDR) algorithm in R. Potential batch effect between sequencing runs were considered as a possible confounding factor when performing multiple comparisons throughout the study (Additional file 19: Table S8) and have been treated as an extra sample variable along with other sample variables for FDR-multiple comparison using “p.adjust” in R. Where indicated in the main text, post-hoc KW pairwise comparison tests for significance between individual groups were performed using the kruskalmc function in R package pgirmess (http:// cran.r-project.org/web/packages/pgirmess/index.html), following significant KW observations (adjusted *p* ≤ 0.05). Spearman’s and Kendall’s *τ* ranked correlations were computed using the “cor.test” function in R.

## Additional files

Additional file 1: Figure S1. Heat maps based on relative abundance (RA) of top genera across four seasons within each individual, grouped by anatomical site. Top genera with average RA of ≥1% in the dataset are represented. Note the different relative abundance ranges provided for each genus. Individuals are color-coded on the y-axis according to their households. (PDF 6992 kb)

Additional file 2: Table S1. Relative abundance and coefficients of variations of top genera. Top genera with average relative abundance of ≥1% across the dataset are represented. For each genus, individual, and skin site, the CV is represented by the standard deviation of the relative abundance of that genus divided by its mean relative abundance over the four seasons. (XLSX 322 kb)

Additional file 3: Figure S2. Coefficient of variation (CV) measurements based on relative abundance (RA) of top genera on a) forehead, b) left and c) right forearm, and d) left and e) right palm sites for each individual. CV is indicated by color gradient, and RA for each individual, site, and genus indicated by point size. Top genera are those with average RA of ≥1% in the dataset. Individuals are color-coded according to households. (PDF 7184 kb)

Additional file 4: Figure S3. Correlation between standard deviation and average relative abundance of genera grouped by individuals. Standard deviation and average relative abundance of the 100 most abundant genera are plotted, with Spearman’s ρ correlation significant for all individuals (adjusted-*p* < 0.05 for all). Each point represents a particular genus found on either forehead (red), forearm (green), or palm (blue) sites within each individual. Spearman’s correlation and linear regression line calculated and constructed in R. (PDF 2767 kb)

Additional file 5: Table S2. Deseq2 and negative binomial mixed model analyses of OTUs showing differential abundance between season pairs. (XLSX 203 kb)

Additional file 6: Figure S4. Cohort-wide seasonal differences in relative proportions of oligotypes for top genera. Oligotypes 2, 1, 1, and 1 of a) *Sphingomonas*, b) *Bacillus*, c) *Propionibacterium*, and d) *Staphylococcus*, respectively, show cohort-wide differences in relative abundance of different oligotypes at specific seasons across cohort and skin sites. At the same time, oligotypes 3 and 4, 9, and 5 of e) *Enhydrobacter*, f) *Sphingomonas*, and g) *Chryseobacterium*, respectively, show household-specific differences in relative abundance of different oligotypes at different seasons across cohort and skin sites. All relative abundance comparisons statistically significant between seasons or households (KW *p* < 0.05 for all oligotypes focused). (PDF 7042 kb)

Additional file 7: Table S3. Shannon diversity across sites, seasons, and individuals, and diversity comparison between sample groups. Shannon diversity calculated based on “breakaway” package in R [56]. (XLSX 51 kb)

Additional file 8: Figure S5. Coefficient of variation (CV) of Shannon diversity between a) individuals, b) households, and c) age groups, grouped by anatomical sites. The Shannon diversity CV of particular skin microbiota is represented by sizes of circles. For a) individuals are color-coded to represent the households as shown in b). (PDF 1863 kb)

Additional file 9: Table S4. Community composition, seasonal, and time-decay analyses. (XLSX 35 kb)

Additional file 10: Figure S6. Density plots of pairwise weighted UniFrac distances between samples of the same (red) and different (blue) seasons. Density plots faceted according to each individual. Only within-individual pairwise comparisons were included in analysis. (PDF 3915 kb)

Additional file 11: Figure S7. Correlations between the pairwise weighted UniFrac within-site distances and Shannon diversity CV of skin sites over time within individuals. Spearman’s correlation and linear regression determined and constructed in R. *P*-values adjusted using false-discovery rate method. (PDF 2015 kb)

Additional file 12: Figure S8. Microbial association network for each individual over the period of one year. Significant correlative associations between OTUs (represented by nodes) within each individual determined based on the SPIEC-EASI pipeline. Correlations between OTUs can be positive (represented by blue edges) or negative (represented by red edges). SPIEC-EASI correlations with magnitude of < 0.05 are not represented in figure. OTUs belonging to one of top 20 taxonomic families are color-coded, whereas OTUs of other families are grouped into “Minor/Unclassified” group, represented by dark gray nodes. (PDF 5774 kb)

Additional file 13: Table S5. Significant SPIEC-EASI correlations between pairs of OTUs and taxonomic information of hub OTUs. Absolute correlations of ≥0.05 are included in the table. (XLSX 232 kb)

Additional file 14: Figure S9. SPEIC-EASI density plot of positive and negative correlations involving OTUs of the same (orange shade) or different (purple shade) genera, and heatmap plots of pairwise significant correlations of OTUs of the top genera within each individual. Only significant correlations with an absolute SPIEC-EASI correlation magnitude of ≥0.05 are included. (PDF 6984 kb)

Additional file 15: Figure S10. Network structure properties per individual over four seasons. a) Node degree distribution is plotted for each individual for winter (blue), spring (green), summer (red), and autumn (black). In households with multiple occupants, distributions from each individual are combined into single plots. b-c) Natural connectivity of microbial association network of each individual in winter (blue), spring (green), summer (red), and autumn (black) upon sequential node removal in order of decreasing b) node betweenness centrality (i.e. nodes having the shortest paths to other nodes removed first) and c) node degree (i.e. nodes having the highest number of edges to other nodes removed first). Natural connectivity, as a measure of a network’s robustness and stability to node removal, is plotted against removal of up to 80% of nodes of a given network. Natural connectivity is expressed as the relative proportion of the size of the original network prior to node removal. (PDF 6137 kb)

Additional file 16: Table S6. Pairwise weighted UniFrac distances between samples from the same individual, cohabitants, and non-cohabitants. (XLSX 49 kb)

Additional file 17: Figure S11. Effect of household occupancy on the extents of changes in skin microbiota within individuals over time. Kendall’s correlation between household occupancy and CVs of relative abundance for a) *Propionibacterium* and b) *Acinetobacter* on forehead, c) *Acinetobacter* on forearm, and d) *Bacillus* on palm within an individual. Correlation between household occupancy and e) Shannon diversity CV and f) pairwise weighted UniFrac distances between communities on the same individual and site over any two seasons. Kendall’s correlation and linear regression determined and constructed in R. Significant correlations following false-discovery rate adjustment are in bold (adjusted-*p <* 0.05). (PDF 4782 kb)

Additional file 18: Table S7. Sample metadata. (XLSX 73 kb)

Additional file 19: Table S8. Weighted UniFrac distance Global R analysis for effect of sequencing batch on microbial community composition within a season. (DOCX 43 kb)

## References

[1] Belkaid Y, Tamoutounour S (2016). The influence of skin microorganisms on cutaneous immunity. Nat Rev Immunol.

[2] Oh J, Freeman AF, Park M, Sokolic R, Candotti F, Holland SM (2013). The altered landscape of the human skin microbiome in patients with primary immunodeficiencies. Genome Res.

[3] Hanski I, von Hertzen L, Fyhrquist N, Koskinen K, Torppa K, Laatikainen T (2012). Environmental biodiversity, human microbiota, and allergy are interrelated. Proc Natl Acad Sci U S A.

[4] Xu Z, Wang Z, Yuan C, Liu X, Yang F, Wang T (2016). Dandruff is associated with the conjoined interactions between host and microorganisms. Sci Rep.

[5] Leung MHY, Wilkins D, Lee PKH (2015). Insights into the pan-microbiome: skin microbial communities of Chinese individuals differ from other racial groups. Sci Rep.

[6] Leung MHY, Chan KCK, Lee PKH (2016). Skin fungal community and its correlation with bacterial community of urban Chinese individuals. Microbiome..

[7] van Rensburg JJ, Lin H, Gao X, Toh E, Fortney KR, Ellinger S (2015). The human skin microbiome associates with the outcome of and is influenced by bacterial infection. MBio.

[8] Findley K, Oh J, Yang J, Conlan S, Deming C, Meyer JA (2013). Topographic diversity of fungal and bacterial communities in human skin. Nature.

[9] Oh J, Byrd AL, Deming C, Conlan S, Kong HH, NISC Comparative Sequencing Program (2014). Biogeography and individuality shape function in the human skin metagenome. Nature.

[10] Oh J, Byrd AL, Park M, Kong HH, Segre JA (2016). Temporal stability of the human skin microbiome. Cell.

[11] Perez GIP, Gao Z, Jourdain R, Ramirez J, Gany F, Clavaud C (2016). Body site is a more determinant factor than human population diversity in the healthy skin microbiome. PLoS One.

[12] Meadow JF, Bateman AC, Herkert KM, O’Connor TK, Green JL (2013). Significant changes in the skin microbiome mediated by the sport of roller derby. PeerJ.

[13] Clemente JC, Pehrsson EC, Blaser MJ, Sandhu K, Gao Z, Wang B (2015). The microbiome of uncontacted Amerindians. Sci Adv.

[14] Blaser MJ, Dominguez-Bello MG, Contreras M, Magris M, Hidalgo G, Estrada I (2013). Distinct cutaneous bacterial assemblages in a sampling of South American Amerindians and US residents. ISME J..

[15] Hospodsky D, Pickering AJ, Julian TR, Miller D, Gorthala S, Boehm AB (2014). Hand bacterial communities vary across two different human populations. Microbiology.

[16] Grice EA, Kong HH, Conlan S, Deming CB, Davis J, Young AC (2009). Topographical and temporal diversity of the human skin microbiome. Science.

[17] Costello EK, Lauber CL, Hamady M, Fierer N, Gordon JI, Knight R (2009). Bacterial community variation in human body habitats across space and time. Science.

[18] Kong HH, Oh J, Deming C, Conlan S, Grice EA, Beatson MA (2012). Temporal shifts in the skin microbiome associated with disease flares and treatment in children with atopic dermatitis. Genome Res.

[19] Abeles SR, Jones MB, Santiago-Rodriguez TM, Ly M, Klitgord N, Yooseph S (2016). Microbial diversity in individuals and their household contacts following typical antibiotic courses. Microbiome..

[20] Flores GE, Caporaso JG, Henley JB, Rideout JR, Domogala D, Chase J (2014). Temporal variability is a personalized feature of the human microbiome. Genome Biol.

[21] Wilkins D, Leung MHY, Lee PKH (2017). Microbiota fingerprints lose individually identifying features over time. Microbiome..

[22] Faust K, Sathirapongsasuti JF, Izard J, Segata N, Gevers D, Raes J (2012). Microbial co-occurrence relationships in the human microbiome. PLoS Comput Biol.

[23] Song SJ, Lauber C, Costello EK, Lozupone CA, Humphrey G, Berg-Lyons D (2013). Cohabiting family members share microbiota with one another and with their dogs. elife.

[24] Ly M, Jones MB, Abeles SR, Santiago-Rodriguez TM, Gao J, Chan IC (2016). Transmission of viruses via our microbiomes. Microbiome..

[25] Fierer N, Hamady M, Lauber CL, Knight R (2008). The influence of sex, handedness, and washing on the diversity of hand surface bacteria. Proc Natl Acad Sci U S A.

[26] Ling Z, Liu X, Luo Y, Yuan L, Nelson KE, Wang Y (2013). Pyrosequencing analysis of the human microbiota of healthy Chinese undergraduates. BMC Genomics.

[27] Zeeuwen PL, Boekhorst J, van den Bogaard EH, de Koning HD, van de Kerkhof PM, Saulnier DM (2012). Microbiome dynamics of human epidermis following skin barrier disruption. Genome Biol.

[28] Mukherjee S, Mitra R, Maitra A, Gupta S, Kumaran S, Chakrabortty A (2016). Sebum and hydration levels in specific regions of human face significantly predict the nature and diversity of facial skin microbiome. Sci Rep.

[29] Bouslimani A, Porto C, Rath CM, Wang M, Guo Y, Gonzalez A (2015). Molecular cartography of the human skin surface in 3D. Proc Natl Acad Sci U S A.

[30] Ying S, Zeng DN, Chi L, Tan Y, Galzote C, Cardona C (2015). The influence of age and gender on skin-associated microbial communities in urban and rural human populations. PLoS One.

[31] Staudinger T, Pipal A, Redl B (2011). Molecular analysis of the prevalent microbiota of human male and female forehead skin compared to forearm skin and the influence of make-up. J Appl Microbiol.

[32] Wilkins D, Leung MHY, Lee PKH (2016). Indoor air bacterial communities in Hong Kong households assemble independently of occupant skin microbiomes. Environ Microbiol.

[33] Lax S, Smith DP, Hampton-Marcell J, Owens SM, Handley KM, Scott NM (2014). Longitudinal analysis of microbial interaction between humans and the indoor environment. Science.

[34] Fitz-Gibbon S, Tomida S, Chiu B-H, Nguyen L, Du C, Liu M (2013). *Propionibacterium acnes* strain populations in the human skin microbiome associated with acne. J Invest Dermatol.

[35] N’Diaye AR, Leclerc C, Kentache T, Hardouin J, Poc CD, Konto-Ghiorghi Y (2016). Skin-bacteria communication: involvement of the neurohormone calcitonin gene related peptide (CGRP) in the regulation of *Staphylococcus epidermidis* virulence. Sci Rep.

[36] Shade A, Gregory Caporaso J, Handelsman J, Knight R, Fierer N (2013). A meta-analysis of changes in bacterial and archaeal communities with time. ISME J..

[37] Maurice CF, Knowles S CL, Ladau J, Pollard KS, Fenton A, Pedersen AB (2015). Marked seasonal variation in the wild mouse gut microbiota. ISME J..

[38] Davenport ER, Mizrahi-Man O, Michelini K, Barreiro LB, Ober C, Gilad Y (2014). Seasonal variation in human gut microbiome composition. PLoS One.

[39] Kurtz ZD, Müller CL, Miraldi ER, Littman DR, Blaser MJ, Bonneau RA (2015). Sparse and compositionally robust inference of microbial ecological networks. PLoS Comput Biol.

[40] Faust K, Lima-Mendez G, Lerat J-S, Sathirapongsasuti JF, Knight R, Huttenhower C (2015). Cross-biome comparison of microbial association networks. Syst Microbiol.

[41] Shade A, Jones SE, Caporaso JG, Handelsman J, Knight R, Fierer N (2014). Conditionally rare taxa disproportionately contribute to temporal changes in microbial diversity. mBio.

[42] Layeghifard M, Hwang DM, Guttman DS (2017). Disentangling interactions in the microbiome: a network perspective. Trends Microbiol.

[43] Christensen GJM, Scholz CFP, Enghild J, Rohde H, Kilian M, Thürmer A (2016). Antagonism between *Staphylococcus epidermidis* and *Propionibacterium acnes* and its genomic basis. BMC Genomics.

[44] Wollenberg MS, Claesen J, Escapa IF, Aldridge KL, Fischbach MA, Lemon KP (2014). Propionibacterium-produced coproporphyrin III induces Staphylococcus aureus aggregation and biofilm formation. MBio.

[45] Ward MJ, Goncheva M, Richardson E, McAdam PR, Raftis E, Kearns A (2016). Identification of source and sink populations for the emergence and global spread of the East-Asia clone of community-associated MRSA. Genome Biol.

[46] Ma B, Wang H, Dsouza M, Lou J, He Y, Dai Z (2016). Geographic patterns of co-occurrence network topological features for soil microbiota at continental scale in eastern China. ISME J.

[47] Knights D, Kuczynski J, Charlson ES, Zaneveld J, Mozer MC, Collman RG (2011). Bayesian community-wide culture-independent microbial source tracking. Nat Methods.

[48] Leung MHY, Lee PKH (2016). The roles of the outdoors and occupants in contributing to a potential pan-microbiome of the built environment: a review. Microbiome..

[49] Ruiz-Calderon JF, Cavallin H, Song SJ, Novoselac A, Pericchi LR, Hernandez JN (2016). Walls talk: microbial biogeography of homes spanning urbanization. Sci Adv.

[50] Halfvarson J, Brislawn CJ, Lamendella R, Vázquez-Baeza Y, Walters WA, Bramer LM (2017). Dynamics of the human gut microbiome in inflammatory bowel disease. Nat Microbiol.

[51] Leung MHY, Wilkins D, Li EKT, Kong FKF, Lee PKH (2014). Indoor-air microbiome in an urban subway network: diversity and dynamics. Appl Environ Microbiol.

[52] Edgar RC (2010). Search and clustering orders of magnitude faster than BLAST. Bioinformatics.

[53] Edgar RC (2013). UPARSE: highly accurate OTU sequences from microbial amplicon reads. Nat Methods.

[54] Caporaso JG, Kuczynski J, Stombaugh J, Bittinger K, Bushman FD, Costello EK (2010). QIIME allows analysis of high-throughput community sequencing data. Nat Methods.

[55] Edgar R (2016). UCHIME2: improved chimera prediction for amplicon sequencing. bioRxiv.

[56] Willis A, Bunge J (2014). Estimating diversity via frequency ratios. Biometrics.

[57] Eren AM, Maignien L, Sul WJ, Murphy LG, Grim SL, Morrison HG (2013). Oligotyping: differentiating between closely related microbial taxa using 16S rRNA gene data. Methods Ecol Evol.

[58] Mahana D, Trent CM, Kurtz ZD, Bokulich NA, Battaglia T, Chung J (2016). Antibiotic perturbation of the murine gut microbiome enhances the adiposity, insulin resistance, and liver disease associated with high-fat diet. Genome Med.

[59] Morris A, Paulson JN, Talukder H, Tipton L, Kling H, Cui L (2016). Longitudinal analysis of the lung microbiota of cynomolgous macaques during long-term SHIV infection. Microbiome.

[60] Liu H, Roeder K, Wasserman L. Stability approach to regularization selection (StARS) for high dimensional graphical models. Adv Neural Inf Process Syst. 2010;24:1432–40. PMC4138724 25152607

[61] Shannon P, Markiel A, Ozier O, Baliga NS, Wang JT, Ramage D (2003). Cytoscape: a software environment for integrated models of biomolecular interaction networks. Genome Res.

[62] Yaveroğlu ÖN, Malod-Dognin N, Davis D, Levnajic Z, Janjic V, Karapandza R (2014). Revealing the hidden language of complex networks. Sci Rep.

