# Supplementary figures and images for: Individual and household attributes influence the dynamics of the personal skin microbiota and its association network
Source: Microbiome. 2018 Feb 2;6:26. doi: 10.1186/s40168-018-0412-9 (PMC5797343; doi:10.1186/s40168-018-0412-9)

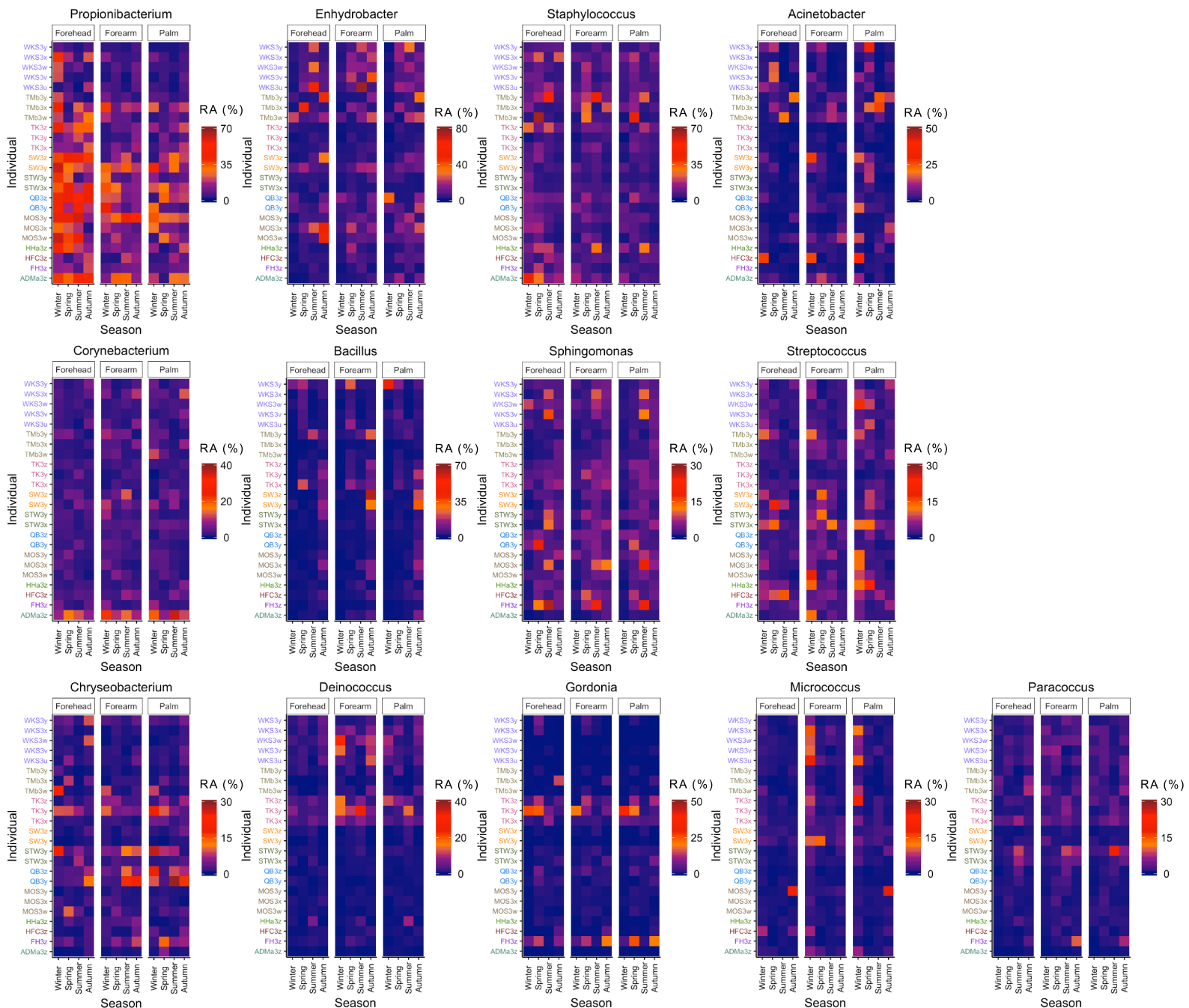

Additional File 1: Figure S1

Supplement: Supplementary file 1 — Heat maps based on relative abundance (RA) of top genera across four seasons within each individual, grouped by anatomical site. Top genera with average RA of ≥1% in the dataset are represented. Note the different relative abundance ranges provided for each genus. Individuals are color-coded on the y-axis according to their households. (PDF 6992 kb) [file 40168_2018_412_MOESM1_ESM.pdf]

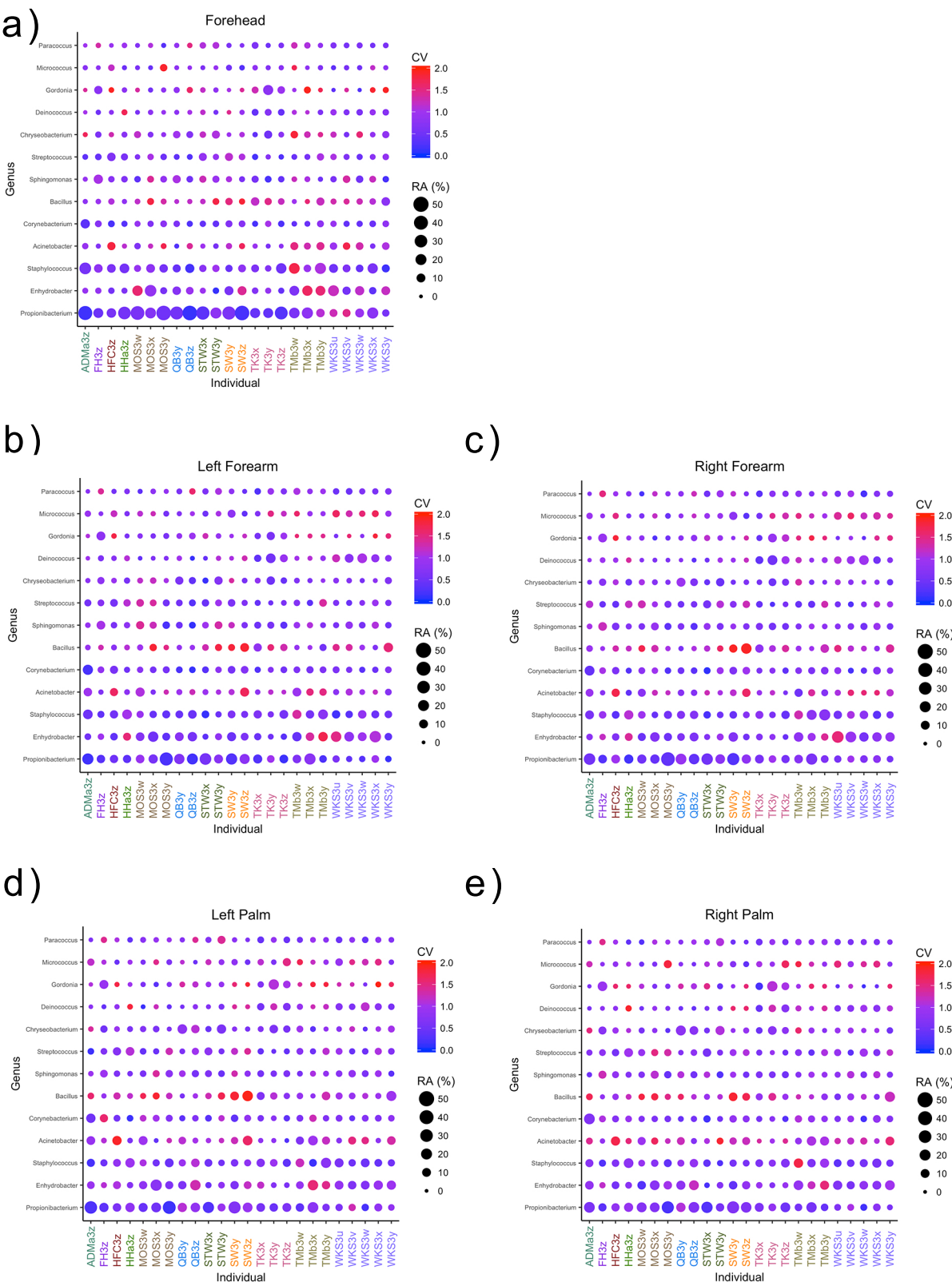

Additional File 3: Figure S2

Supplement: Supplementary file 3 — Coefficient of variation (CV) measurements based on relative abundance (RA) of top genera on a) forehead, b) left and c) right forearm, and d) left and e) right palm sites for each individual. CV is indicated by color gradient, and RA for each individual, site, and genus indicated by point size. Top genera are those with average RA of ≥1% in the dataset. Individuals are color-coded according to households. (PDF 7184 kb) [file 40168_2018_412_MOESM3_ESM.pdf]

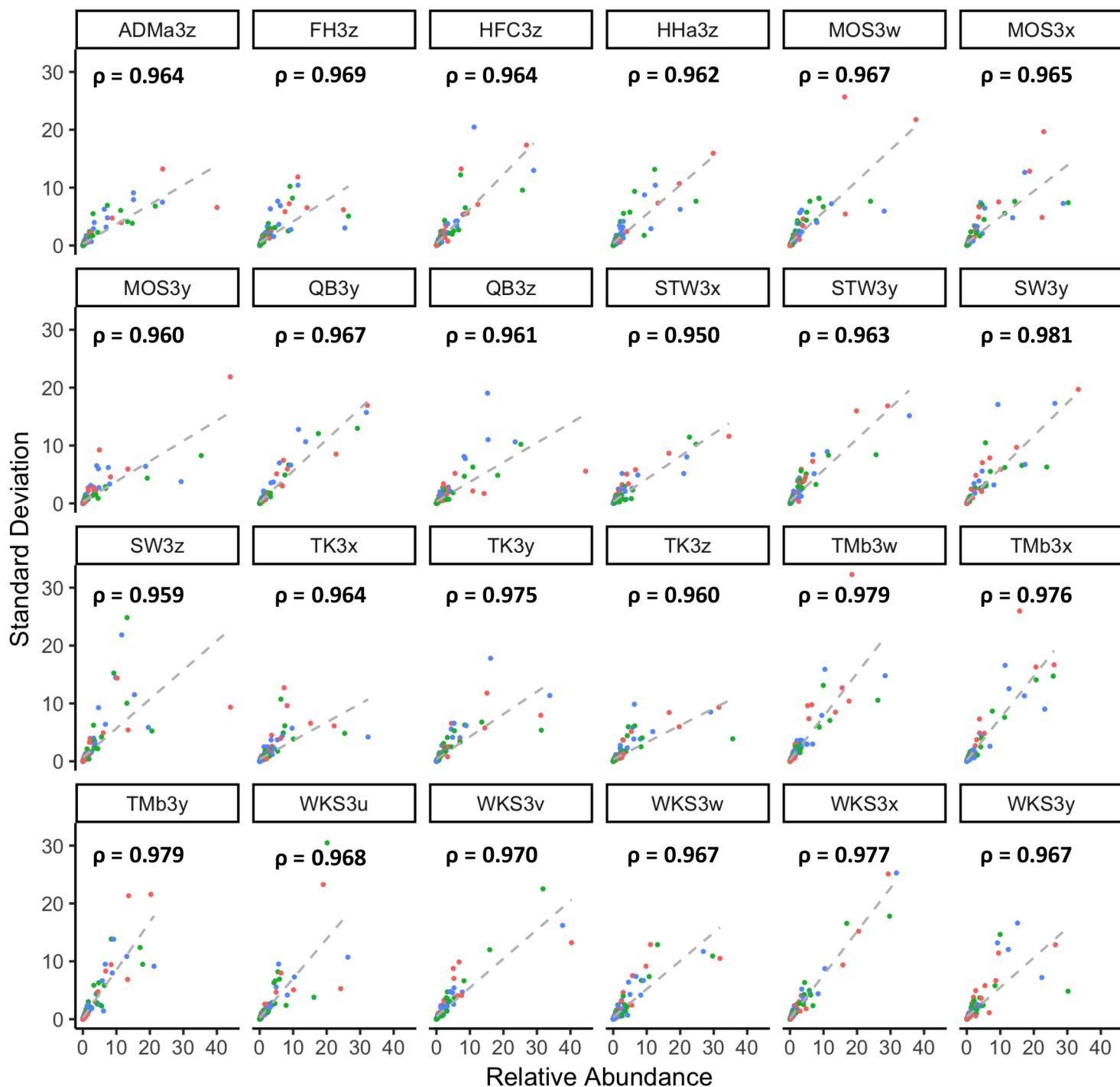

Supplement: Supplementary file 4 — Correlation between standard deviation and average relative abundance of genera grouped by individuals. Standard deviation and average relative abundance of the 100 most abundant genera are plotted, with Spearman’s ρ correlation significant for all individuals (adjusted-p < 0.05 for all). Each point represents a particular genus found on either forehead (red), forearm (green), or palm (blue) sites within each individual. Spearman’s correlation and linear regression line calculated and constructed in R. (PDF 2767 kb) [file 40168_2018_412_MOESM4_ESM.pdf]

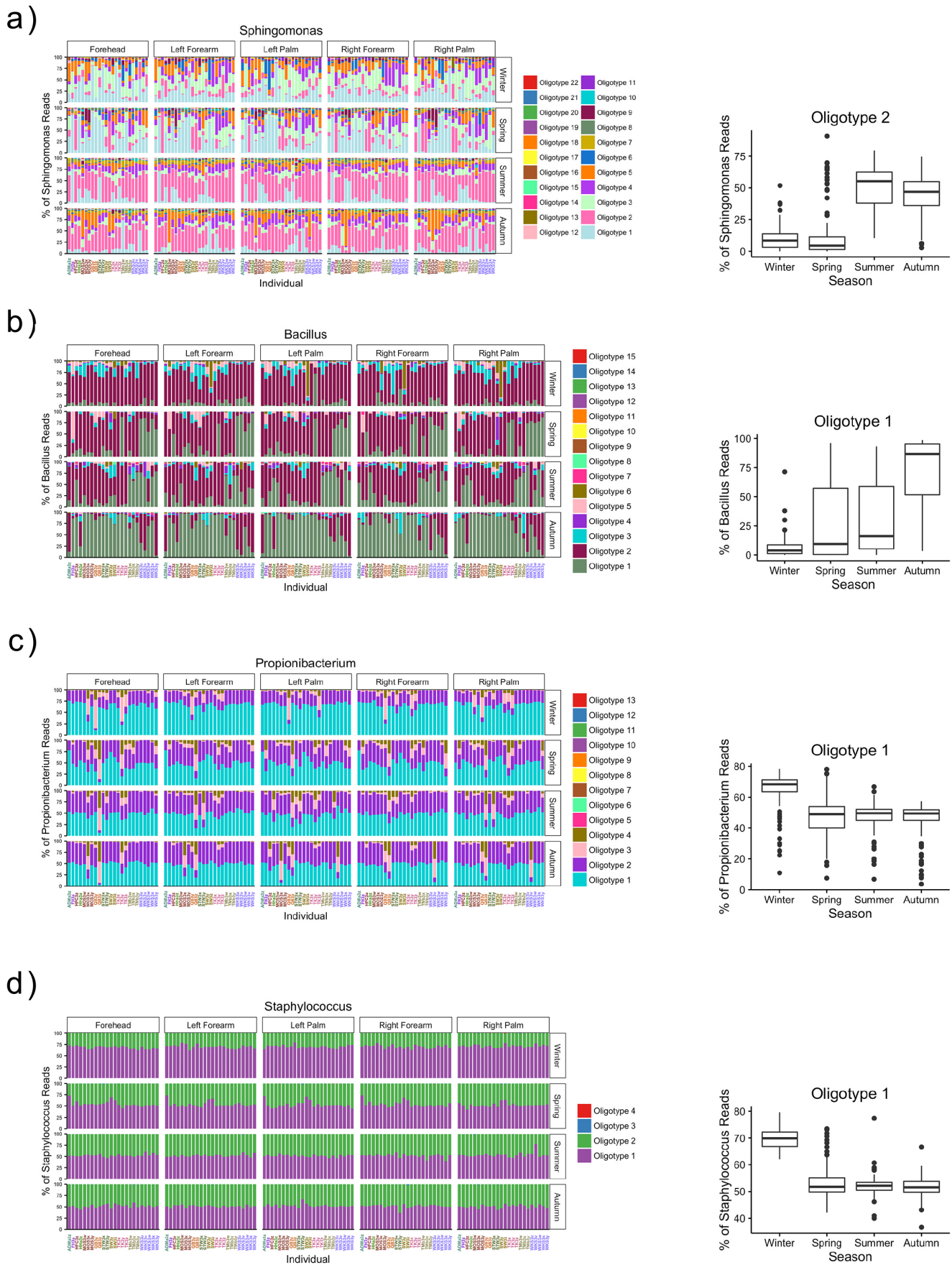

Additional File 6: Figure S4

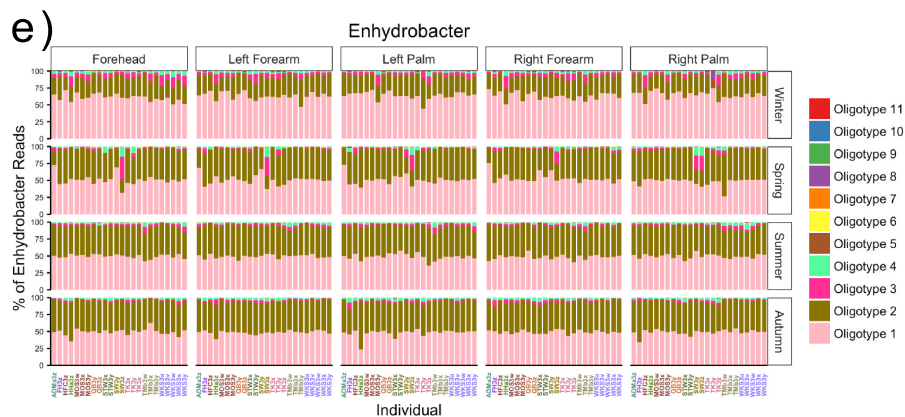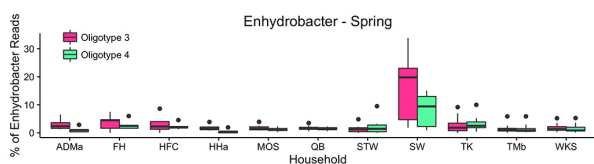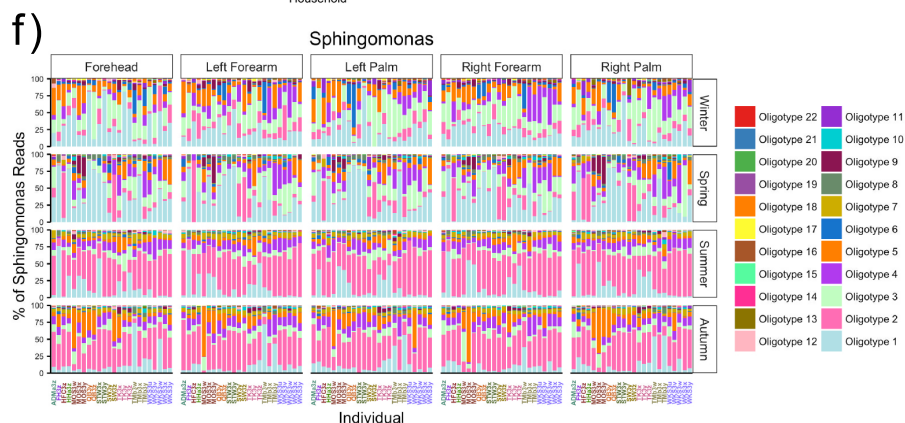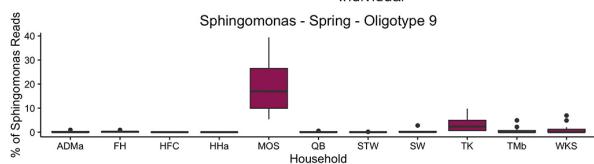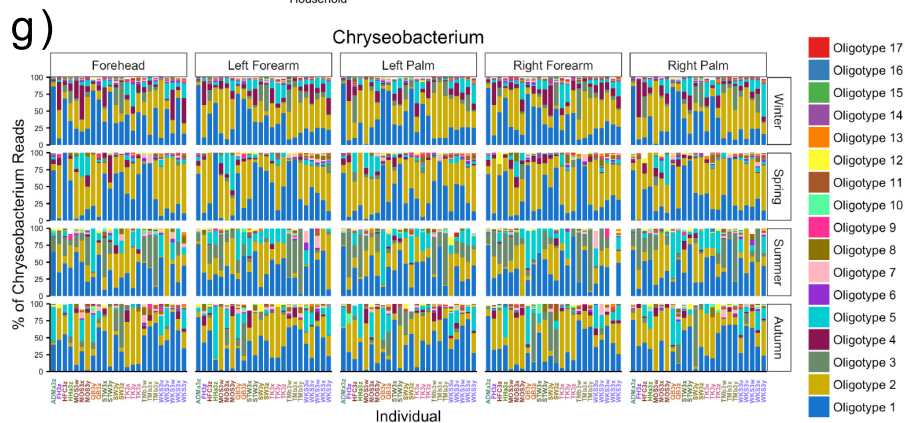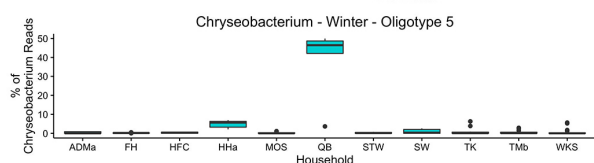

Supplement: Supplementary file 6 — Cohort-wide seasonal differences in relative proportions of oligotypes for top genera. Oligotypes 2, 1, 1, and 1 of a) Sphingomonas, b) Bacillus, c) Propionibacterium, and d) Staphylococcus, respectively, show cohort-wide differences in relative abundance of different oligotypes at specific seasons across cohort and skin sites. At the same time, oligotypes 3 and 4, 9, and 5 of e) Enhydrobacter, f) Sphingomonas, and g) Chryseobacterium, respectively, show household-specific differences in relative abundance of different oligotypes at different seasons across cohort and skin sites. All relative abundance comparisons statistically significant between seasons or households (KW p < 0.05 for all oligotypes focused). (PDF 7042 kb) [file 40168_2018_412_MOESM6_ESM.pdf]

a)

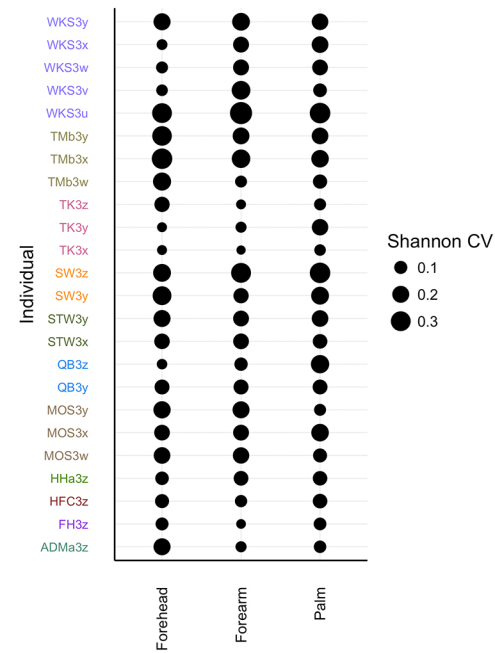

b)

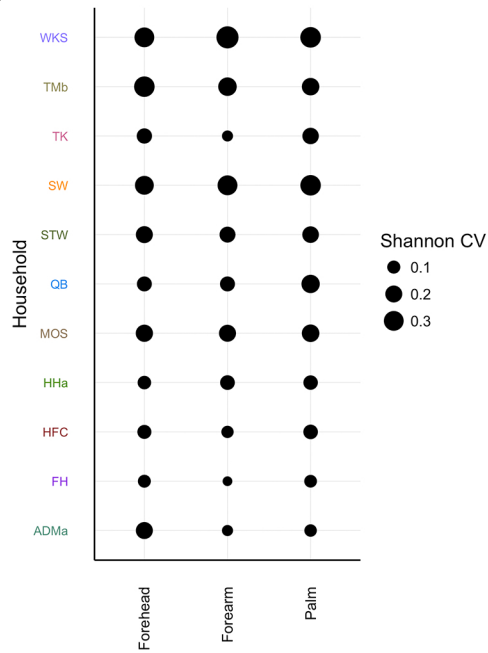

c)

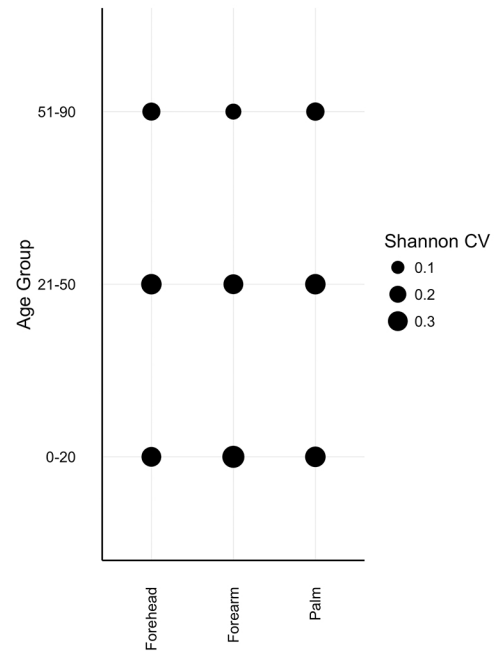

Supplement: Supplementary file 8 — Coefficient of variation (CV) of Shannon diversity between a) individuals, b) households, and c) age groups, grouped by anatomical sites. The Shannon diversity CV of particular skin microbiota is represented by sizes of circles. For a) individuals are color-coded to represent the households as shown in b). (PDF 1863 kb) [file 40168_2018_412_MOESM8_ESM.pdf]

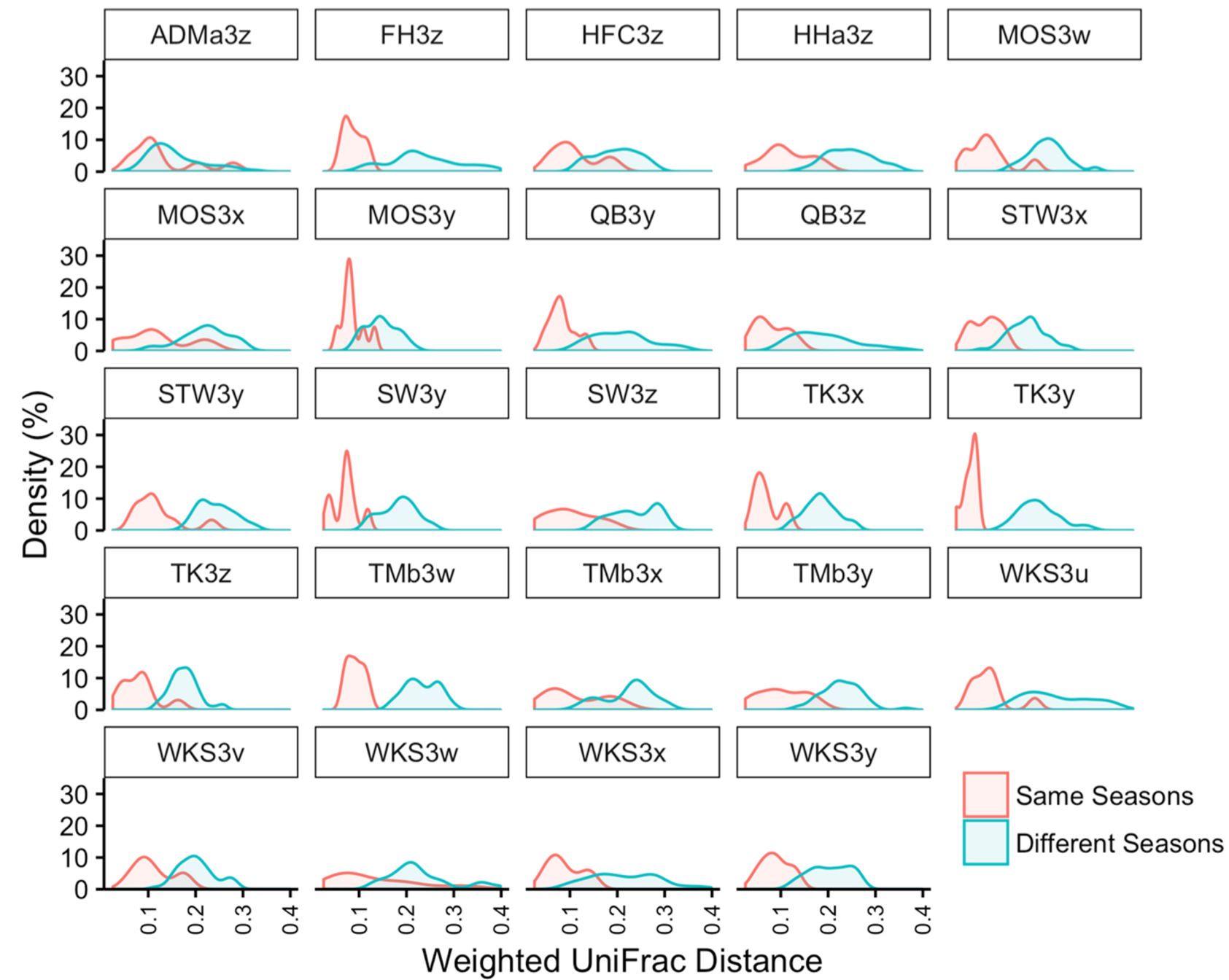

Additional File 10: Figure S6

Supplement: Supplementary file 10 — Density plots of pairwise weighted UniFrac distances between samples of the same (red) and different (blue) seasons. Density plots faceted according to each individual. Only within-individual pairwise comparisons were included in analysis. (PDF 3915 kb) [file 40168_2018_412_MOESM10_ESM.pdf]

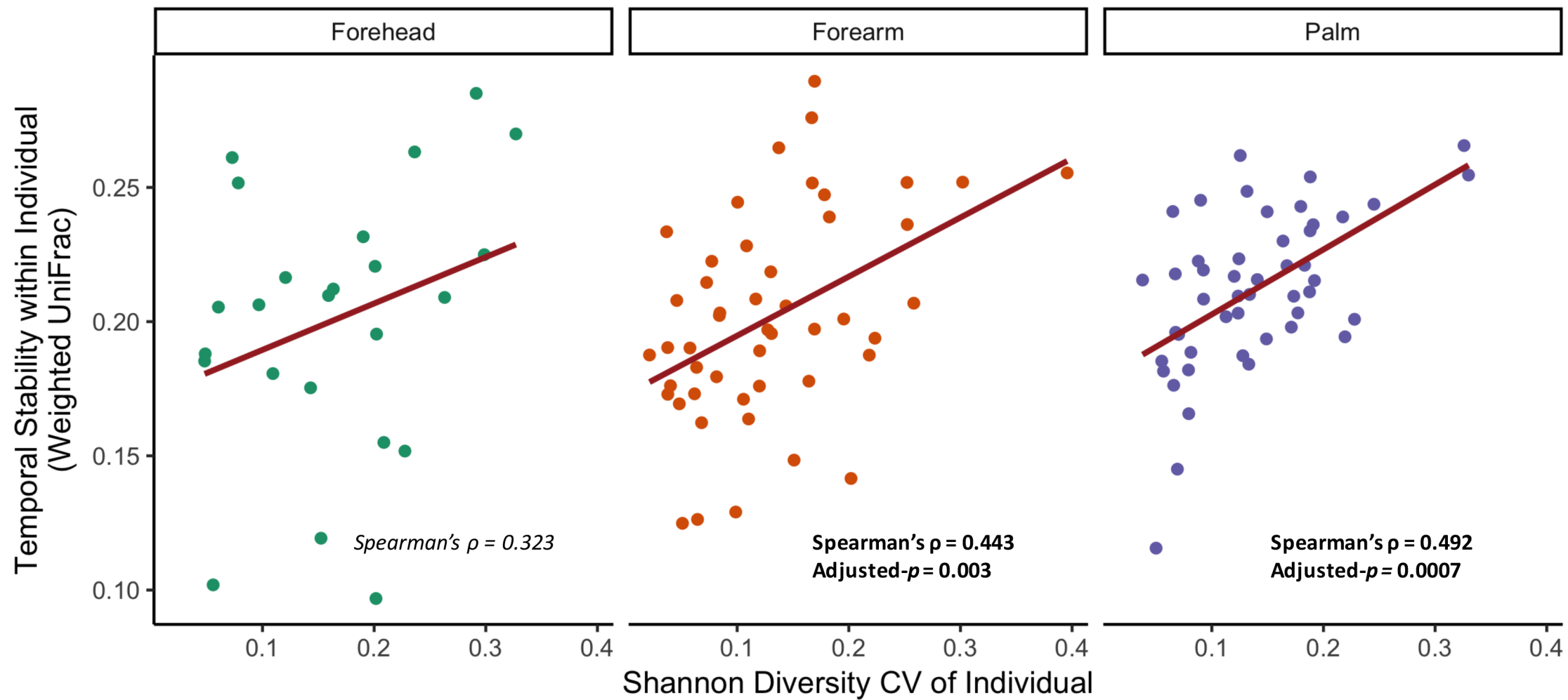

Additional File 11: Figure S7

Supplement: Supplementary file 11 — Correlations between the pairwise weighted UniFrac within-site distances and Shannon diversity CV of skin sites over time within individuals. Spearman’s correlation and linear regression determined and constructed in R. P-values adjusted using false-discovery rate method. (PDF 2015 kb) [file 40168_2018_412_MOESM11_ESM.pdf]

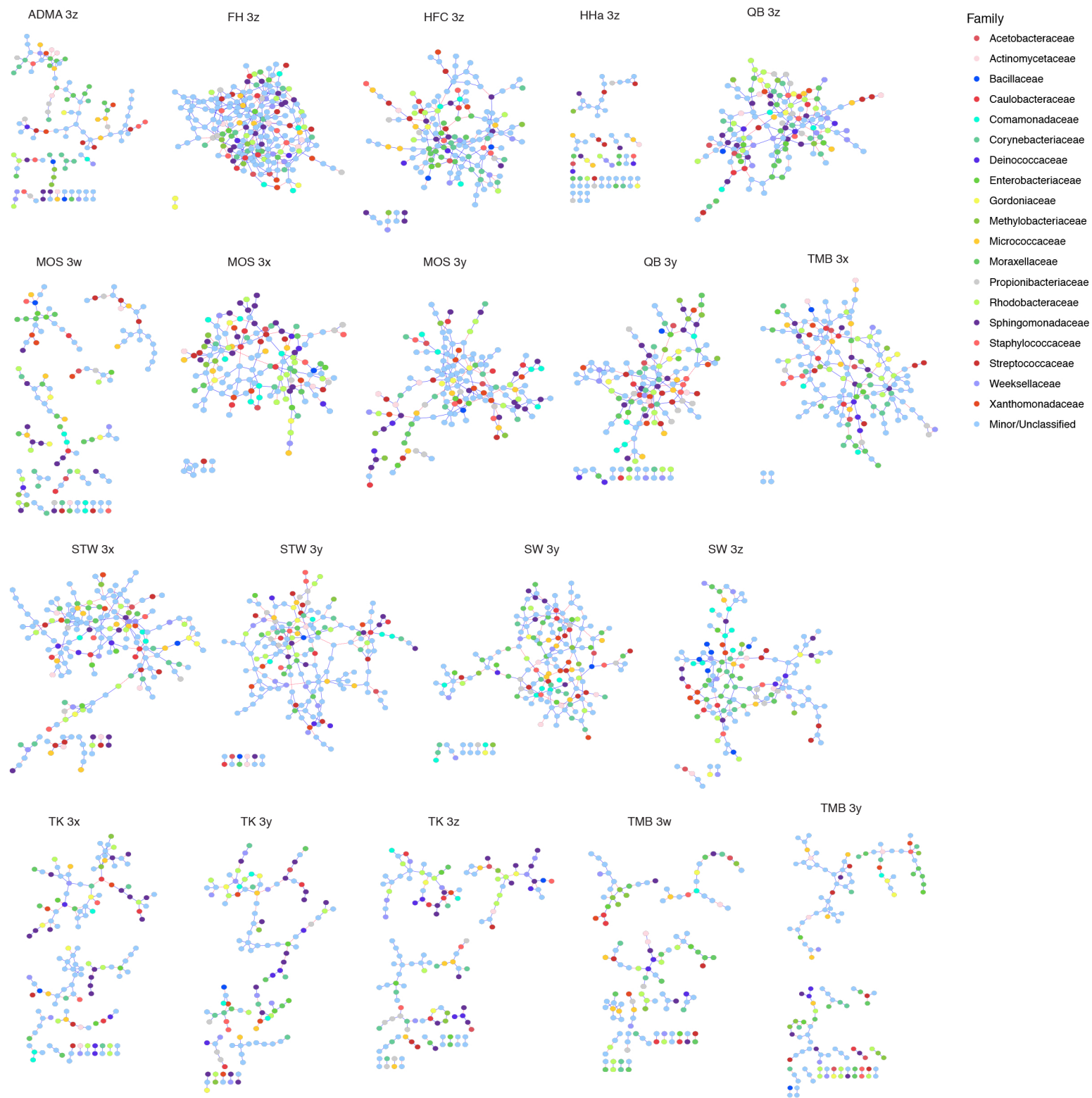

Additional File 12: Figure S8

Supplement: Supplementary file 12 — Microbial association network for each individual over the period of one year. Significant correlative associations between OTUs (represented by nodes) within each individual determined based on the SPIEC-EASI pipeline. Correlations between OTUs can be positive (represented by blue edges) or negative (represented by red edges). SPIEC-EASI correlations with magnitude of < 0.05 are not represented in figure. OTUs belonging to one of top 20 taxonomic families are color-coded, whereas OTUs of other families are grouped into “Minor/Unclassified” group, represented by dark gray nodes. (PDF 5774 kb) [file 40168_2018_412_MOESM12_ESM.pdf]

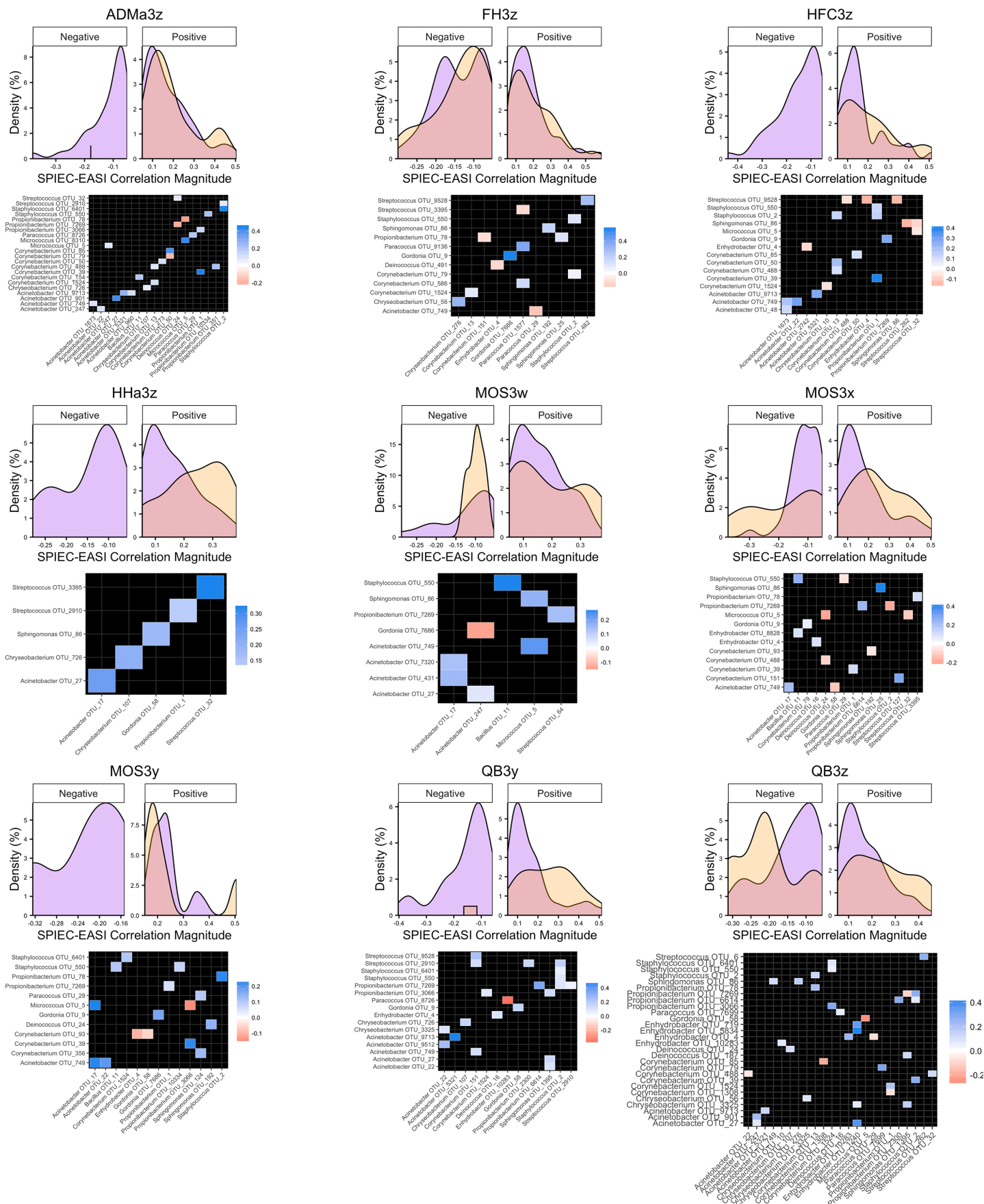

Additional File 14: Figure S9

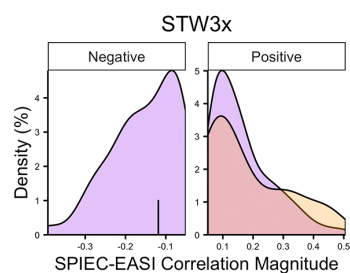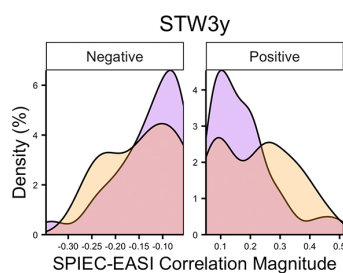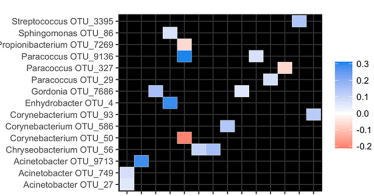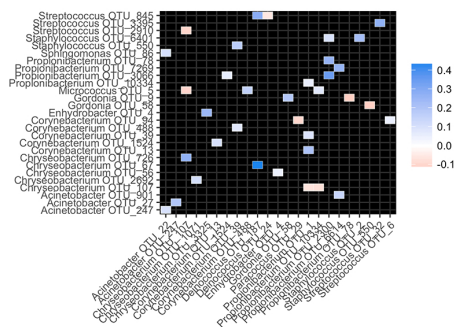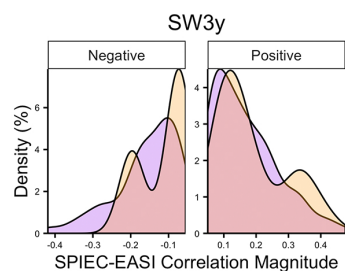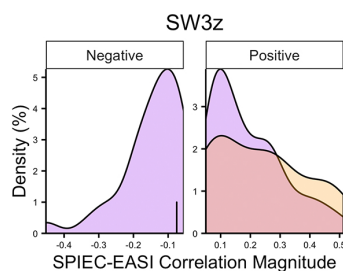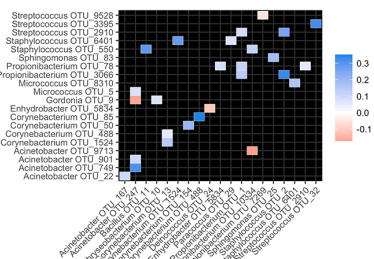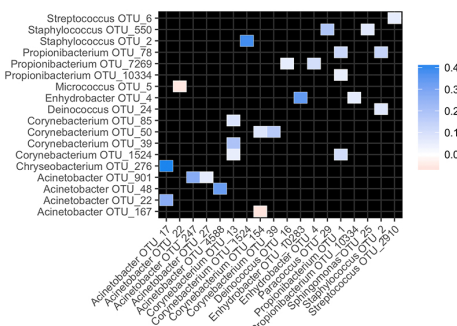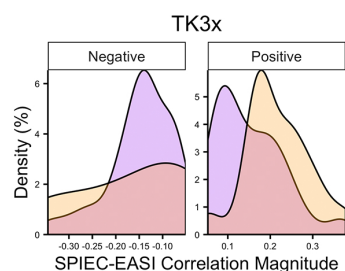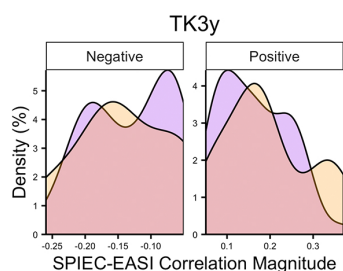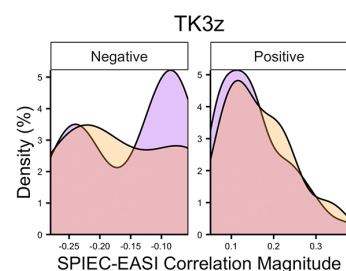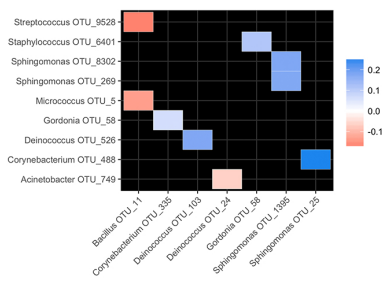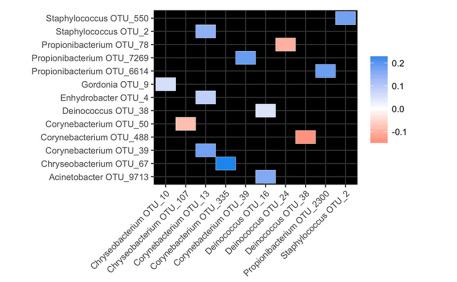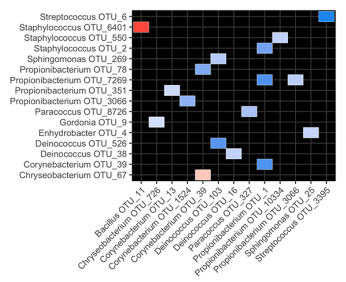

Additional File 14: Figure S9

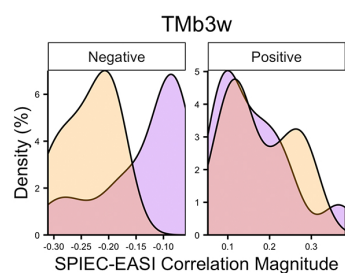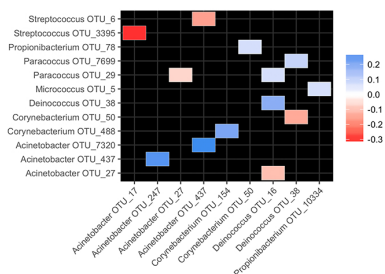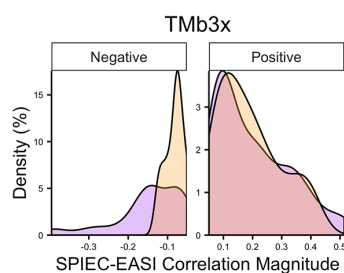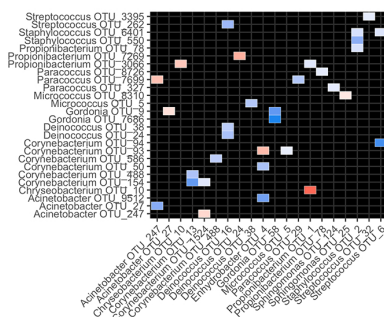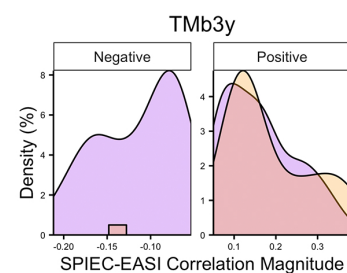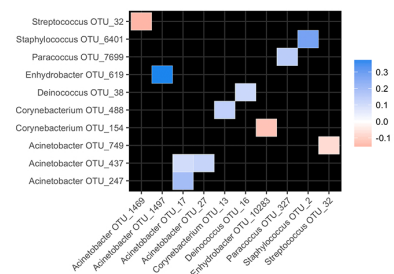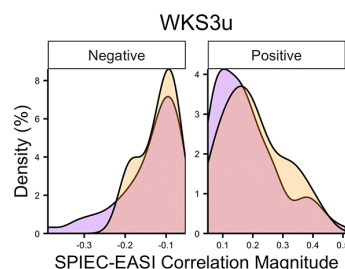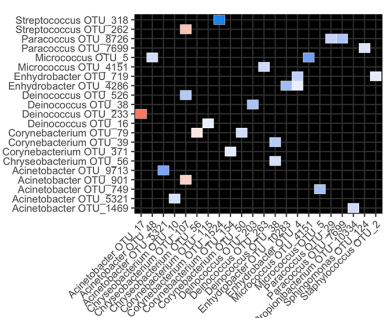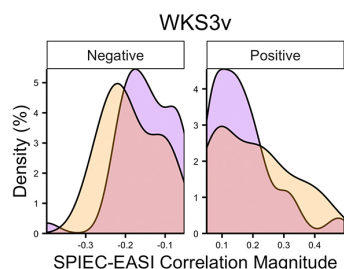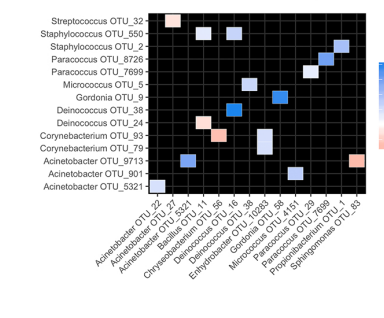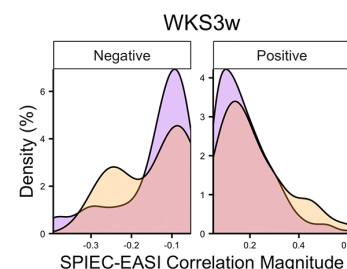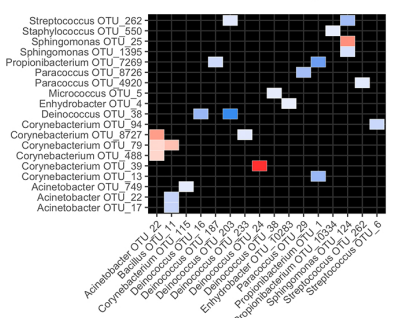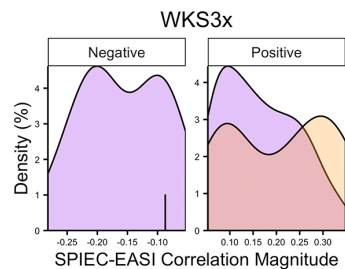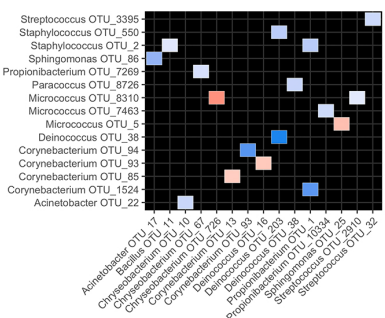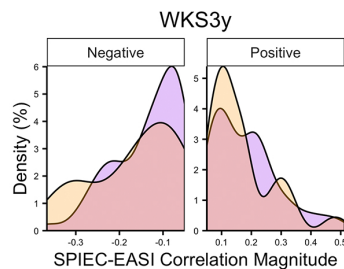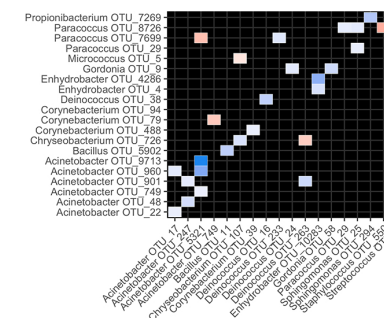

Additional File 14: Figure S9

Supplement: Supplementary file 14 — SPEIC-EASI density plot of positive and negative correlations involving OTUs of the same (orange shade) or different (purple shade) genera, and heatmap plots of pairwise significant correlations of OTUs of the top genera within each individual. Only significant correlations with an absolute SPIEC-EASI correlation magnitude of ≥0.05 are included. (PDF 6984 kb) [file 40168_2018_412_MOESM14_ESM.pdf]

a)

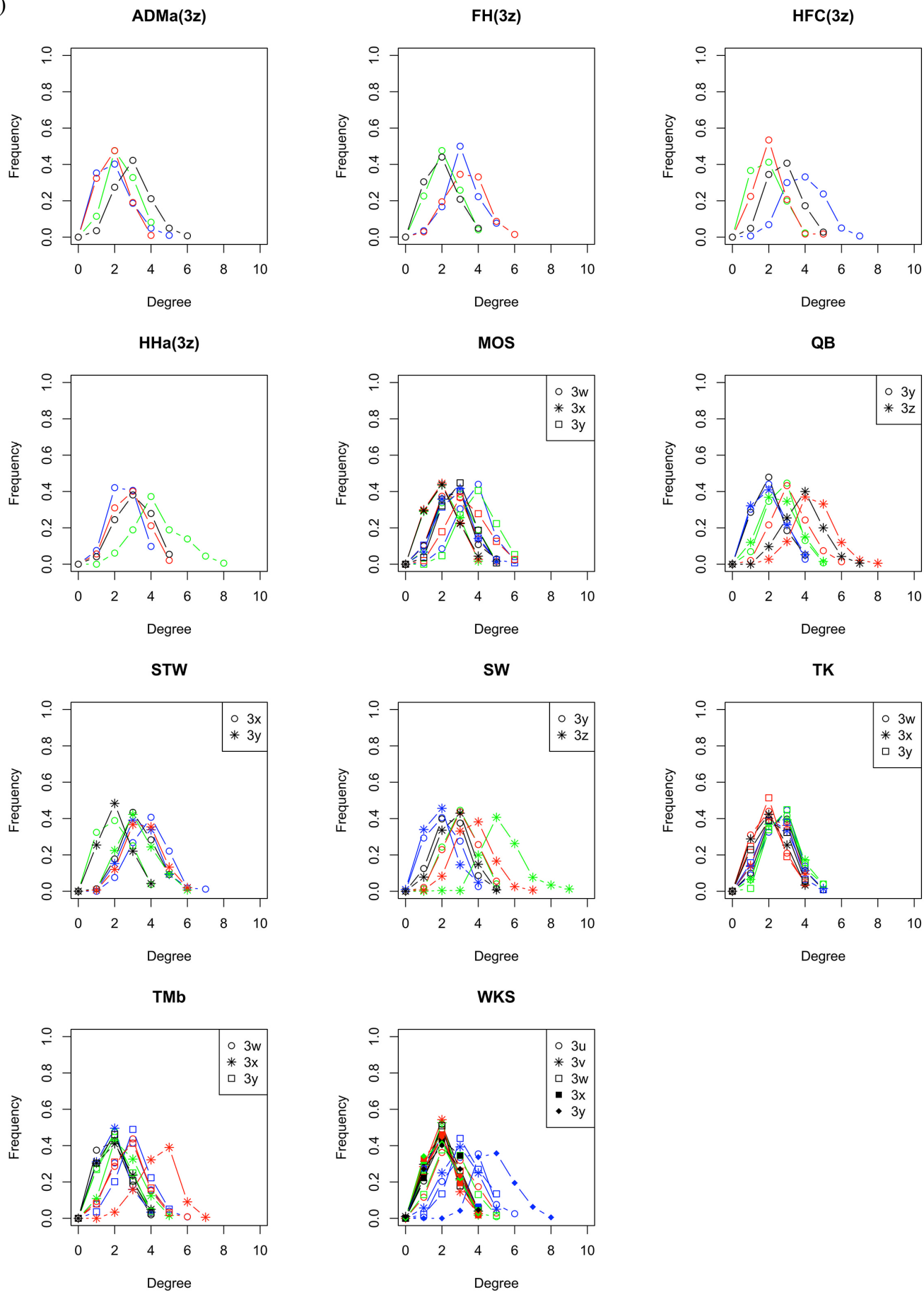

b)

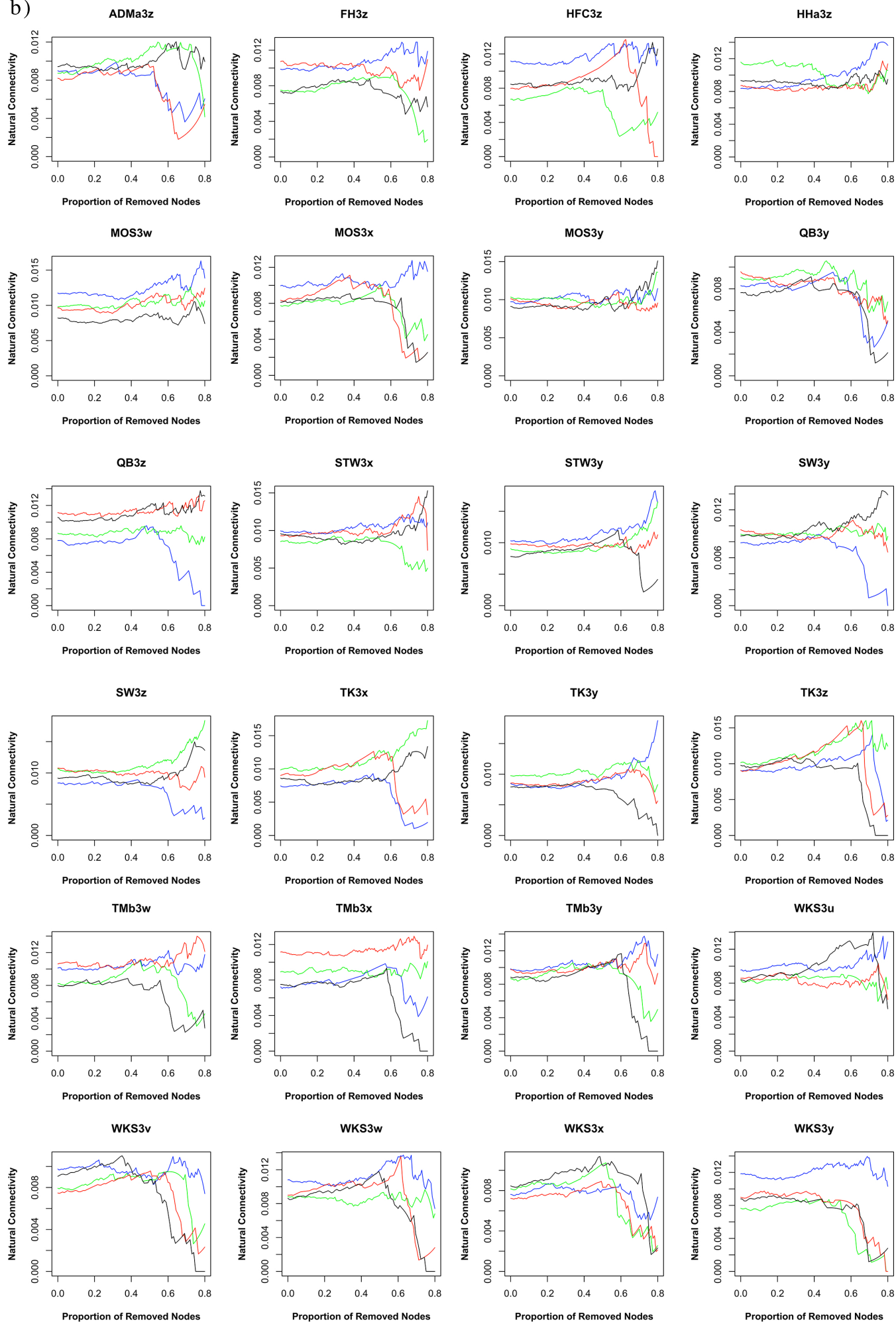

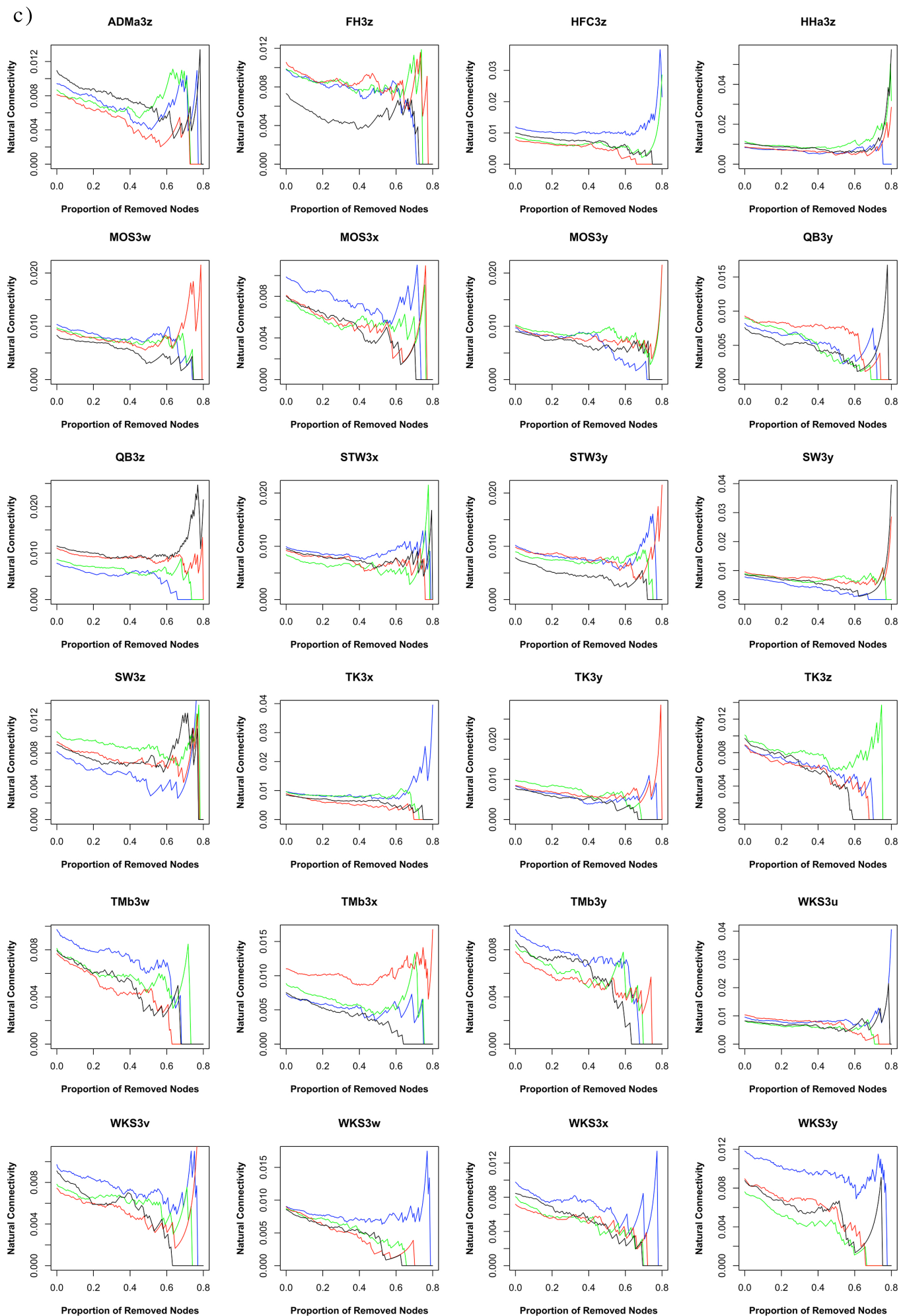

Additional File 15: Figure S10

Supplement: Supplementary file 15 — Network structure properties per individual over four seasons. a) Node degree distribution is plotted for each individual for winter (blue), spring (green), summer (red), and autumn (black). In households with multiple occupants, distributions from each individual are combined into single plots. b-c) Natural connectivity of microbial association network of each individual in winter (blue), spring (green), summer (red), and autumn (black) upon sequential node removal in order of decreasing b) node betweenness centrality (i.e. nodes having the shortest paths to other nodes removed first) and c) node degree (i.e. nodes having the highest number of edges to other nodes removed first). Natural connectivity, as a measure of a network’s robustness and stability to node removal, is plotted against removal of up to 80% of nodes of a given network. Natural connectivity is expressed as the relative proportion of the size of the original network prior to node removal. (PDF 6137 kb) [file 40168_2018_412_MOESM15_ESM.pdf]

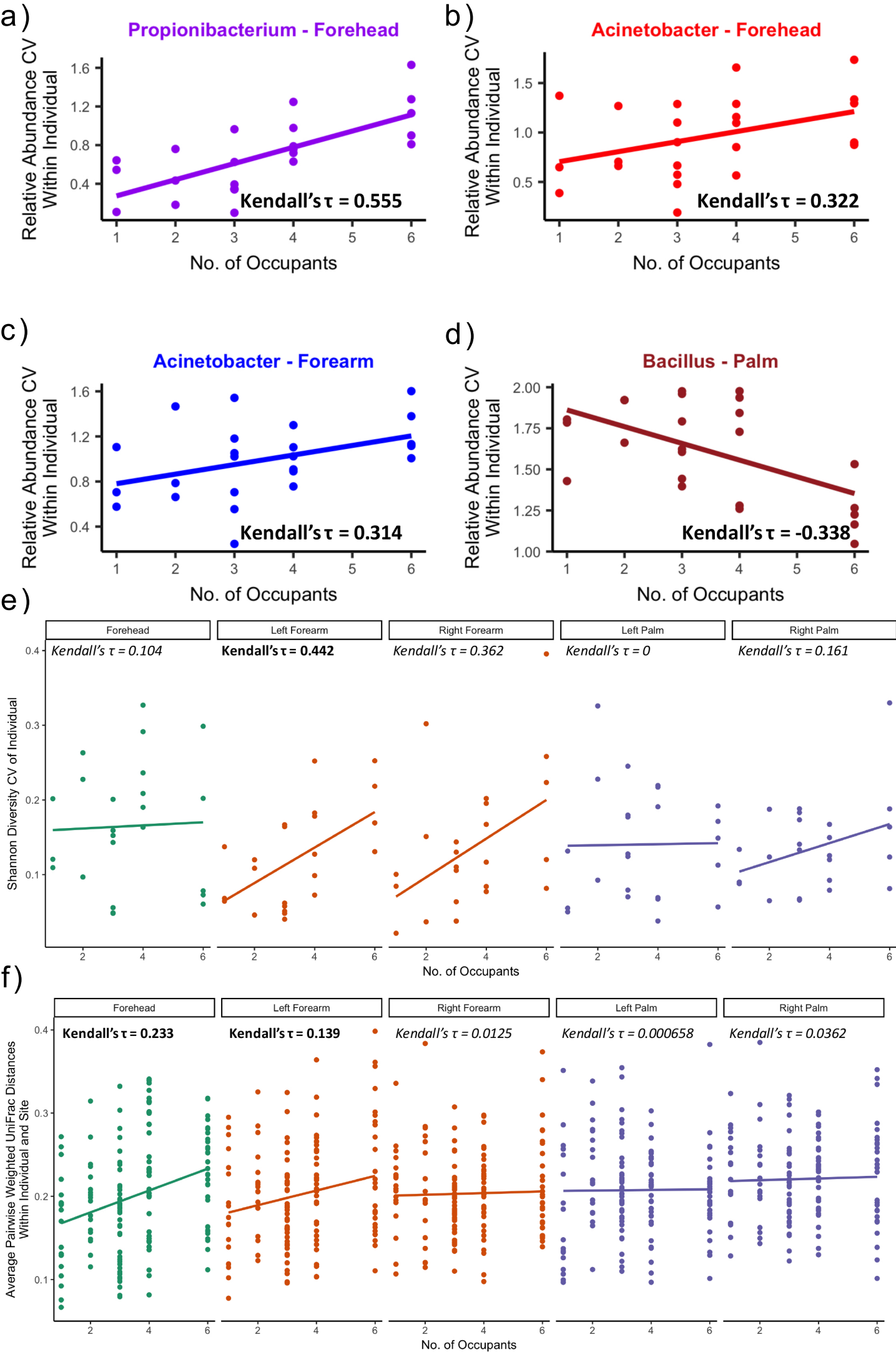

Supplement: Supplementary file 17 — Effect of household occupancy on the extents of changes in skin microbiota within individuals over time. Kendall’s correlation between household occupancy and CVs of relative abundance for a) Propionibacterium and b) Acinetobacter on forehead, c) Acinetobacter on forearm, and d) Bacillus on palm within an individual. Correlation between household occupancy and e) Shannon diversity CV and f) pairwise weighted UniFrac distances between communities on the same individual and site over any two seasons. Kendall’s correlation and linear regression determined and constructed in R. Significant correlations following false-discovery rate adjustment are in bold (adjusted-p < 0.05). (PDF 4782 kb) [file 40168_2018_412_MOESM17_ESM.pdf]
