# Supplementary material for: Individual and household attributes influence the dynamics of the personal skin microbiota and its association network
Source: Microbiome. 2018 Feb 2;6:26. doi: 10.1186/s40168-018-0412-9 (PMC5797343; doi:10.1186/s40168-018-0412-9)
Supplement: Supplementary file 19 — Weighted UniFrac distance Global R analysis for effect of sequencing batch on microbial community composition within a season. (DOCX 43 kb) [file 40168_2018_412_MOESM19_ESM.docx]

**Additional File 19: Table S8. Weighted UniFrac distance Global R analysis for effect of sequencing batch on microbial community composition within a season.**

| **Season** | **Number of Batches^a^** | **ANOSIM Global R**^b^ | ***P*-value** |
| --- | --- | --- | --- |
| Winter | 4 | 0.119 | 0.001 |
| Spring | 5 | 0.0814 | 0.001 |
| Summer | 3 | 0.0633 | 0.006 |
| Autumn | 3 | 0.106 | 0.001 |

^a^Number of sequencing batches within each season.

^b^ANOSIM Global R for grouping of samples based on batches within each season.
